# A proteogenomic view of Parkinson’s disease causality and heterogeneity

**DOI:** 10.1038/s41531-023-00461-9

**Published:** 2023-02-11

**Authors:** Sergio Kaiser, Luqing Zhang, Brit Mollenhauer, Jaison Jacob, Simonne Longerich, Jorge Del-Aguila, Jacob Marcus, Neha Raghavan, David Stone, Olumide Fagboyegun, Douglas Galasko, Mohammed Dakna, Bilada Bilican, Mary Dovlatyan, Anna Kostikova, Jingyao Li, Brant Peterson, Michael Rotte, Vinicius Sanz, Tatiana Foroud, Samantha J. Hutten, Mark Frasier, Hirotaka Iwaki, Andrew Singleton, Ken Marek, Karen Crawford, Fiona Elwood, Mirko Messa, Pablo Serrano-Fernandez

**Affiliations:** 1 Translational Medicine Department, Novartis Institutes for Biomedical Research, Basel, Switzerland; 2 Cardiovascular and Metabolism Department, Novartis Institutes for Biomedical Research, Cambridge, MA USA; 3 Department of Neurology, University Medical Center Göttingen, Göttingen, Germany; 4 Genome and Biomarker Sciences, Merck Exploratory Science Center, Cambridge, MA USA; 5 Department of Genetics, Cerevel Therapeutics, Cambridge, MA USA; 6 Department of Neurosciences, University of Southern California, San Diego, La Jolla, CA USA; 7 Neuroscience Department, Novartis Institutes for Biomedical Research, Cambridge, MA USA; 8 Department of Medical and Molecular Genetics, Indiana University School of Medicine, Indianapolis, IN USA; 9 Michael J. Fox Foundation for Parkinson’s Research, New York, NY USA; 10 Laboratory of Neurogenetics, National Institute on Aging, National Institutes of Health, Bethesda, MD USA; 11 Institute for Neurodegenerative Disorders, New Haven, CT USA; 12 Laboratory of Neuroimaging, University of Southern California, Los Angeles, CA USA; 13 Present Address: Moderna Genomics, Cambridge, MA USA; 14 Present Address: Translational Genomics, Discovery Sciences BioPharmaceuticals R&D, AstraZeneca, Gothenburg, Sweden; 15 Present Address: Valo Health, Cambridge, MA USA; 16 Present Address: The Janssen Pharmaceutical Companies of Johnson & Johnson, Cambridge, MA USA

**Keywords:** Parkinson's disease, Predictive markers, Prognostic markers

## Abstract

The pathogenesis and clinical heterogeneity of Parkinson’s disease (PD) have been evaluated from molecular, pathophysiological, and clinical perspectives. High-throughput proteomic analysis of cerebrospinal fluid (CSF) opened new opportunities for scrutinizing this heterogeneity. To date, this is the most comprehensive CSF-based proteomics profiling study in PD with 569 patients (350 idiopathic patients, 65 *GBA* + mutation carriers and 154 *LRRK2* + mutation carriers), 534 controls, and 4135 proteins analyzed. Combining CSF aptamer-based proteomics with genetics we determined protein quantitative trait loci (pQTLs). Analyses of pQTLs together with summary statistics from the largest PD genome wide association study (GWAS) identified 68 potential causal proteins by Mendelian randomization. The top causal protein, GPNMB, was previously reported to be upregulated in the substantia nigra of PD patients. We also compared the CSF proteomes of patients and controls. Proteome differences between *GBA* + patients and unaffected *GBA* + controls suggest degeneration of dopaminergic neurons, altered dopamine metabolism and increased brain inflammation. In the *LRRK2* + subcohort we found dysregulated lysosomal degradation, altered alpha-synuclein processing, and neurotransmission. Proteome differences between idiopathic patients and controls suggest increased neuroinflammation, mitochondrial dysfunction/oxidative stress, altered iron metabolism and potential neuroprotection mediated by vasoactive substances. Finally, we used proteomic data to stratify idiopathic patients into “endotypes”. The identified endotypes show differences in cognitive and motor disease progression based on previously reported protein-based risk scores.Our findings not only contribute to the identification of new therapeutic targets but also to shape personalized medicine in CNS neurodegeneration.

## Introduction

Parkinson’s disease (PD) is the second most prevalent neurodegenerative disorder worlwide^1^. Parkinson’s disease patients experience selective degeneration and loss of dopaminergic neurons in the substantia nigra *pars compacta*. In most cases, PD is classified as idiopathic, but a growing set of genetic variants increase PD risk or accelerate its onset. Many of the identified genes are involved either in mitochondrial or endo-lysosomal biology^2^. The two most common PD risk genes are leucine rich kinase 2 (*LRRK2*) and glucosidase beta acid (*GBA*). Mutations in these genes are linked to ~10% of sporadic cases and up to 30% in specific ethnic subgroups and familial disease^3^. Some of the genetic variants in *LRRK2* and *GBA* have been associated with specific clinical phenotypes^4,5^. For both genes, the pathological mutations are thought to exacerbate the toxicity of alpha-synuclein, which—in an aggregated form—contributes to neuronal death and amplifies the neuroinflammatory response^6–9^.

Clinical heterogeneity of PD has motivated many disease stratification efforts. Some of those have focused on clinical variables, mostly hypothesis-driven, while others have focused on molecular data, mostly hypothesis-free^10^. While carriers of specific mutations may present specific clinical phenotypes^4,5^, patient strata defined by clinical or molecular variables are not easily linked to specific PD risk mutations^11^.

Proteins hold great potential as predictors, causal biomarkers and surrogates of disease progression and/or stratification. However, the biological and pathophysiological complexity of PD, the difficulties of collecting standardized biological samples (especially cerebrospinal fluid; CSF) from large cohorts throughout the course of disease, and the technical limitations of high-throughput proteomic analyses hamper the identification of biomarkers at a proteomic level. The multicenter Parkinson Progression Marker Initiative (PPMI) was initiated to overcome some of these limitations, particularly in terms of number of samples and clinical data^12^. In this collaboration, 1103 baseline (not longitudinal) CSF samples from patients and control participants with known status of *LRRK2* and *GBA* pathogenic variants were analyzed using the SomaScan aptamer-based proteomics platform^13^. In parallel we also looked at the whole genome sequencing data from 804 patients out of the 1103, after quality control (QC). To our knowledge, this is, to date, the largest proteomic and genetic data set for interrogating causal proteins for PD in a neurologically relevant biofluid. Previously reported characterizations of the proteome in CSF of PD paved the way to identify potential biomarkers and help to better understand the etiology of the disease^14,15^. The conclusions reported in those studies were extracted from smaller patient populations. A recent study^16^ has performed a CSF proteomic profiling of over 1700 proteins using mass spectrometry in the PPMI subcohort of *LRRK2* + patients and a subset of PPMI idiopathic patients as well as in an independent set (Harvard Biomarker Study). The overlaps with our findings are considered in the discussion section.

**Fig. 1:**
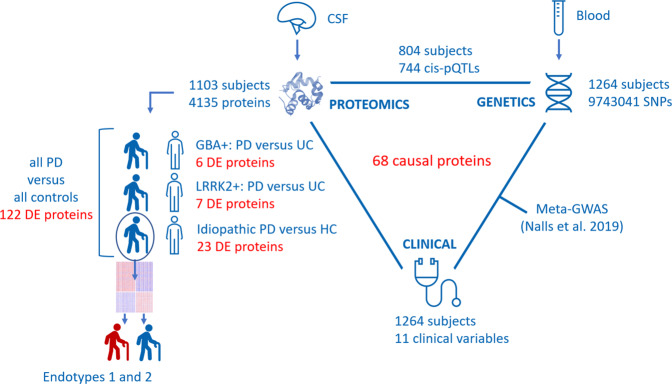
Study analytical design. 1103 subjects were analyzed with SomaScan for 4135 unique proteins in CSF. The comparison between *GBA* + PD patients and *GBA* + unaffected controls (UC) retrieved six differentially expressed (DE) proteins. The comparison between *LRRK2* + PD patients and *LRRK2* + UC retrieved seven DE proteins. The comparison between idiopathic PD patients and HC non-mutation carriers retrieved 23 DE proteins. Patients and controls were also combined and compared, which retrieved 122 DE proteins. Idiopathic PD patients were further analyzed, and two endotypes were identified based on CSF proteomics. 1264 subjects were sequenced genome wide to detect a total of 9743041 SNPs. For the 804 patients that had both genomic and proteomic data, a pQTL analysis was performed that identified 744 unique proteins with a significant cis-pQTL. The pQTLs combined with a meta GWAS for PD performed by Nalls et al.^70^, led to the proposal of 68 unique CSF proteins presumed to be causal for PD.

The main goals of this study are summarized in Fig. 1: i. Parkinson’s disease causal protein identification using mendelian randomization based on proteomics and genetics, ii. identification of differences between PD patients and controls within and between subcohorts (*LRRK2*+, *GBA*+ and idiopathic), and iii. hypothesis-free stratification of idiopathic PD patients into clinically relevant endotypes and then compared by means of protein-based risk scores reflecting cognitive and motor progression^17^.

## Results

### Causal analysis

**Fig. 2:**
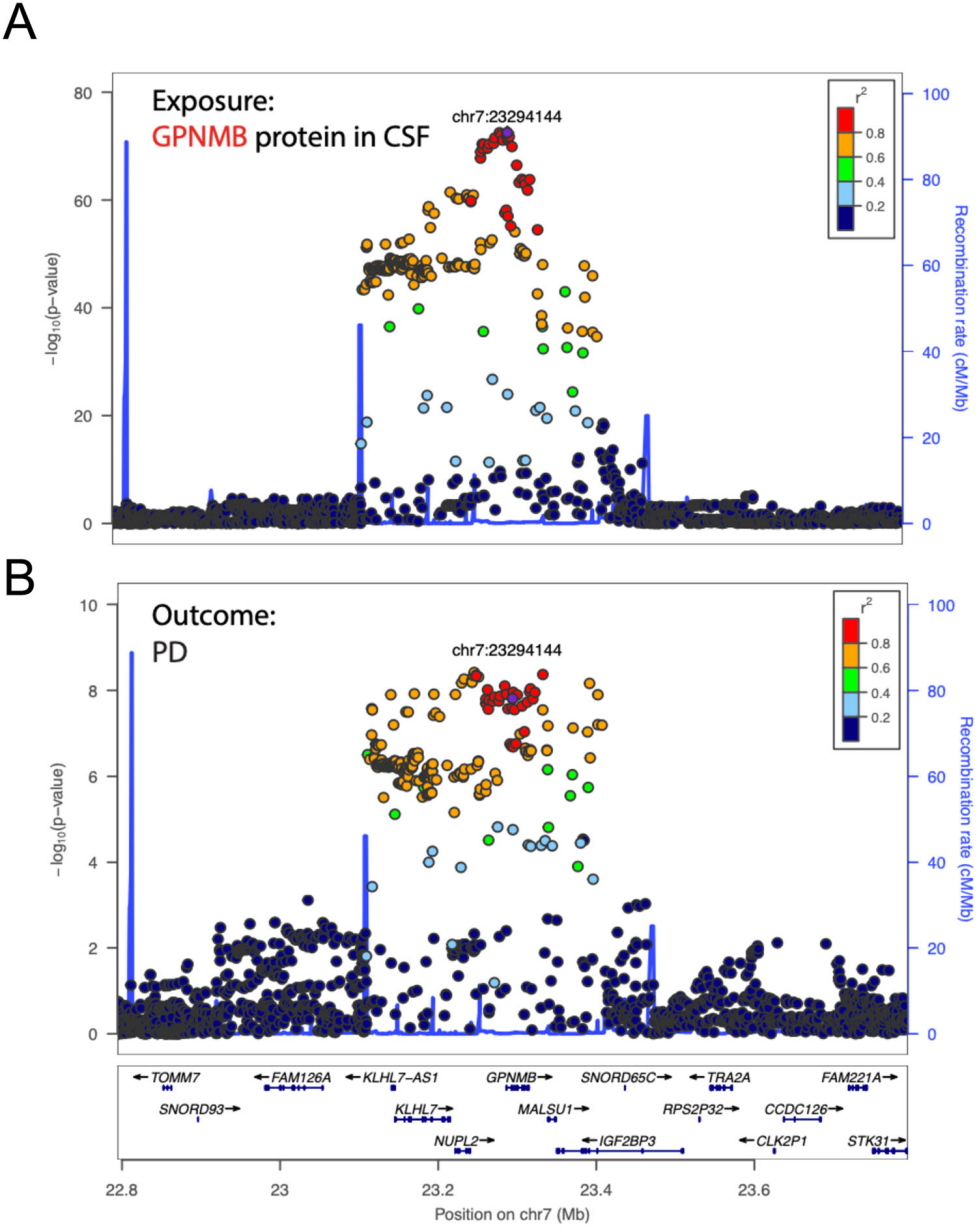
Causal Analysis. **A** Locus visualization of GPNMB pQTL hits suggest a strong association between GPNMB SOMAmer levels and its cis-SNPs. Colors indicate the linkage disequilibrium (LD) correlation of other SNPs with chr7:23294144 (rs858275). **B** Locus visualization of GWAS hits in the GPNMB locus for the risk of developing PD. The *y-*axis is the −log10 nominal *p* value of the GWAS results.

Our analysis reported significant cis-proteomic quantitative trait loci (pQTLs) for 856 Slow Off-rate Modified Aptamers (SOMAmers)—corresponding to 744 unique proteins (Supplementary Table 1). From these 856 cis-pQTLs, we identified statistically significant evidence for causation for 68 proteins in CSF (Table 2). Out of those proteins, GPNMB, FCGR2A and FCGR2B also had a strong colocalization signal (see Methods), indicating the same single nucleotide polymorphism (SNP) is both associated with protein level and PD risk (Fig. 2). The full tables with nominal *P* ≤ 0.05 are included in Supplementary Tables 2 and 3 for less or more stringent clumping, respectively.

### Differential protein expression in subcohorts of Parkinson’s disease patients

To identify proteins differentially expressed in PD, we compared SOMAmers in each of the subcohorts (*GBA*+, *LRRK2*+ and idiopathic) to their corresponding controls. Our statistical analyses revealed six differentially expressed SOMAmers for *GBA* + patients, seven SOMAmers for *LRRK2* + patients and 23 SOMAmers for idiopathic patients. Directionality of the change and adjusted *P* values for each of these markers are reported in Table 3.

For each subcohort, several identified markers confirmed previously reported proteins dysregulated in PD. Interestingly, there was little overlap between proteins dysregulated in *GBA*+, *LRRK2*+ and idiopathic subcohorts (SEMG2 and DLK1 were shared by *GBA*+ and the idiopathic subcohort) though in each list there is a high percentage (4/6 in *GBA*+, 4/7 in *LRRK2*+ and 10/23 in the idiopathic subcohort) of markers previously reported in relation to PD (Table 3).

Only one protein, CTSB, passed the FDR significance threshold for the interaction between disease status and mutation status. As shown in Table 3, CTSB was also differentially expressed in the *LRRK2* + subcohort.

Additional analysis comparing all patient subcohorts with all controls, using subcohort membership (no mutation/*GBA*+/*LRRK2*+) and treatment status (yes/no) as additional covariates resulted in 129 SOMAmers, tagging 122 distinct proteins, passing false discovery rate (FDR) correction (Supplementary Table 4).

### Identification of subtypes of idiopathic Parkinson’s disease patients

#### Identification of endotypes

**Fig. 3:**
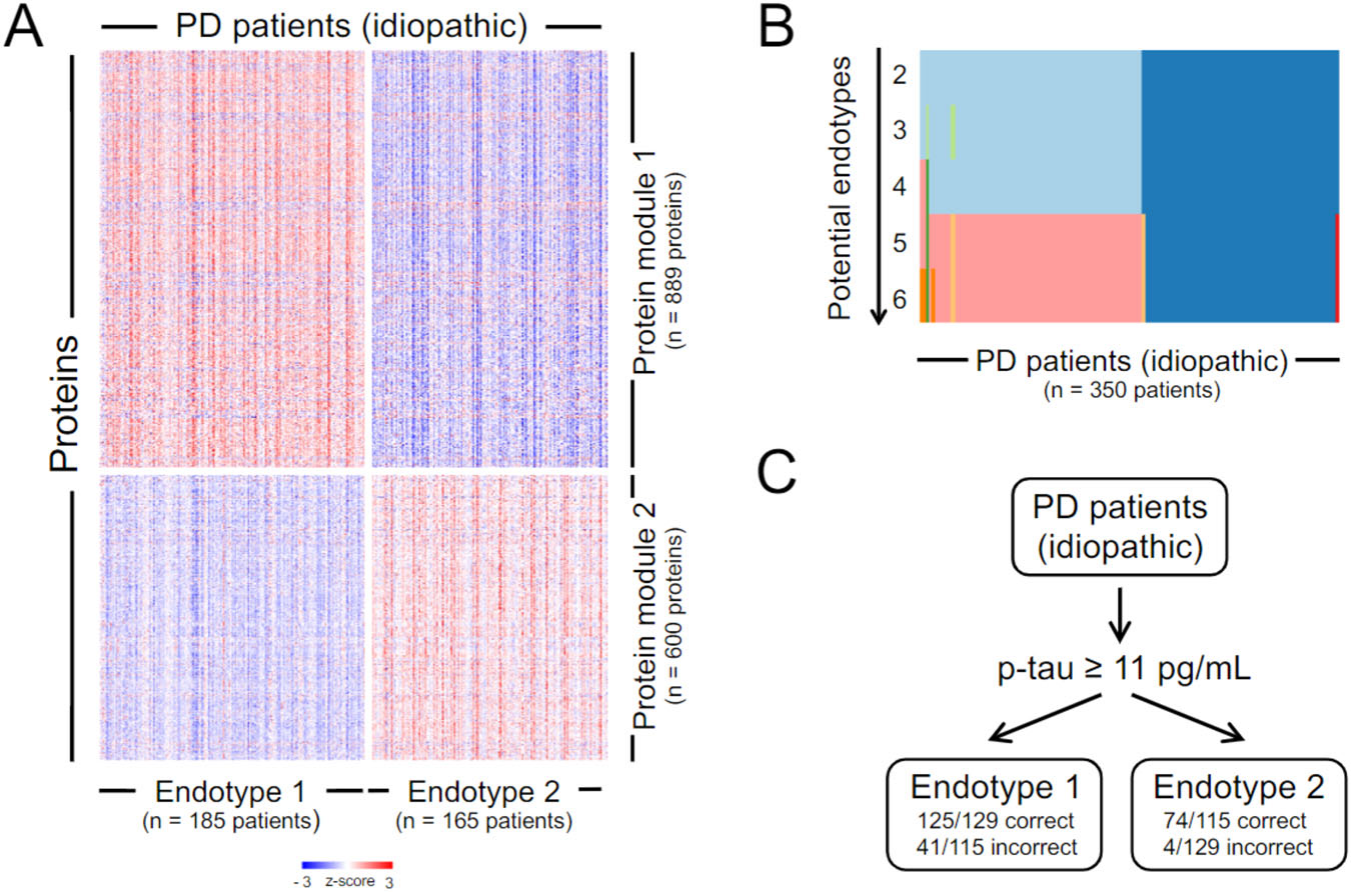
Idiopathic PD endotypes. **A** Heatmap of *z*-scores of the protein values as measured with SomaScan, corresponding to the two modules identified using Weighted Gene Co-expression Network Analysis (WGCNA). The proteins in these modules are used for cluster analysis using Consensus Clustering, which retrieves two clusters (endotypes) of idiopathic PD patients. Patients are shown in the *x*-axis, separated by endotype, while proteins are shown in the *y*-axis, separated by module. **B** Tracking plot depicting how the idiopathic PD patients are assigned to specific endotypes by Consensus Clustering as the number of potential endotypes increases. **C** Partition tree predicting endotype membership of the idiopathic PD patients based on clinical variables only. One node suffices to separate patients into endotypes based on phospho-tau levels (p-tau) in CSF as measured with a clinical assay, the cut-off being 11 pg/mL.

To determine if distinct endotypes were present in the idiopathic subcohort we performed a network analysis on the CSF proteome of idiopathic patients (Weighted Gene Co-expression Network Analysis (WGCNA) block-wise automatic module detection; soft threshold = 11; unsigned TOM, minimum module size = 500, Pearson correlation type). Two modules of co-expressed proteins were identified. They comprised 889 and 600 SOMAmers, respectively. Applying consensus clustering on these two protein modules split the idiopathic subcohort (350 patients) in proteome-based patient endotypes 1 (185 patients) and 2 (165 patients) (Fig. 3A). As seen in the tracking plot (Fig. 3B) these endotypes suffer only negligible changes as the number of modeled subclasses increases. Moreover, a predictive model for the endotypes was built based on clinical parameters, avoiding the re-use of the same proteomic data involved in the definition of the endotypes. Patients with CSF phospho-tau ≥11 pg/mL (as measured with the Elecsys® assay) were enriched for endotype 1, and patients with CSF phospho-tau <11 pg/mL were enriched for endotype 2 (Fig. 3C). The model accuracy in the training (244 patients) and in the independent test (106 patients) sets, was 0.82 and 0.73, respectively. The estimated area under the curve for the test set was 0.77.

Endotypes did not significantly differ in age or sex. There were neither significant differences in caudate, striatum or putamen thickness, nor in UPDRS score parts II and III or MoCA scores. Significant differences were found for UPDRS score part I (higher for endotype 2; *P* = 0.044), as well as CSF levels of amyloid beta (lower for endotype 2; *P* = 1.95 × 10^−15^), phospho-tau (lower for endotype 2; *P* = 1.25 × 10^−15^), total tau (lower for endotype 2; *P* < 2 × 10^−16^) and alpha-synuclein (lower for endotype 2; *P* < 2 × 10^−16^). Endotypes also showed significant differences in “non-genetic Parkinson’s Disease-associated Proteomic Scores” (higher ngPD-ProS for endotype 2; *P* = 0.034).

#### Proteins differentially expressed in endotypes (CSF SomaScan)

To identify the unique proteins significantly dysregulated in each endotype, a linear model was used to identify the differences between each of these two endotypes and the healthy controls to the idiopathic subcohort. For endotype 1, five markers were significantly different compared to the control group (CNTFR, LPO, MMP10, RIPK2, and VEGFA). LPO, RIPK2 and VEGFA were also part of the differences between healthy controls and the whole idiopathic group (see above). Endotype 2, however, showed 200 differentially expressed SOMAmers, 197 unique proteins (see Supplementary Table 5). Among those proteins, AK1, CCL14, FRZB, GPI, HAMP, LPO, NETO1, PTPRR, RAB31, RELT, RIPK2, ROBO3, RSPO4, SHANK1, SPINK9, VEGFA, and VIP were dysregulated for the whole idiopathic group as compared to healthy controls. 155 SOMAmers (i.e., 153 unique proteins; see Supplementary Table 5) were differentially expressed between endotypes.

## Discussion

Independently of its etiology (genetic or idiopathic), multiple cellular and metabolic alterations (e.g., iron metabolism), inflammation, and oxidative stress underly neurodegeneration in PD. In this regard, the identification of causal proteins not only enhances its understanding, but also assists the search for druggable targets. This study identified 68 CSF proteins as causal and GPNMB, FCGR2A, FCGR2B and CTSB were among the top ones.

GPNMB—expressed by myeloid cells—is found at high levels in the substantia nigra of PD patients^18^. Recently, GPNMB has been demonstrated in a cellular model to be necessary and sufficient for the internalization of alpha-synuclein fibrils in the neurons via a functional interaction which would in turn lead to the development of PD. Complementary to that finding, higher GPNMB levels are associated with higher disease risk and higher disease severity^19^. It has also been suggested that GPNMB modulates immune response^20,21^ having primarily a protective role^21–23^. The anti-inflammatory role of GPNMB is mediated by the CD44 non-kinase transmembrane glycoprotein^24^, which has been found to be augmented in PD patients using mass spectrometry in a combination of cohorts from a subset of the PPMI cohort and an independent data set^16^. CTSB cleaves alpha-synuclein fibrils with the potential for decreasing alpha-synuclein aggregation^25^. Both the CTSB locus^26^ and a specific genetic variant of CTSB have been found to be associated with the risk of developing PD^27^. FCGR2A and FCGR2B are involved in phagocytosis and modulate inflammatory responses^28^. Supporting our findings, independent reports suggest that GPNMB, CTSB and FCGR2A are causal to PD^19,29–31^. Alpha-synuclein aggregates bind to the microglia’s FCGR2B receptor, which leads to inhibition of the microglia’s phagocytic activity and indirectly enhances neurodegeneration^32^. In neurons, the FCGR2B receptor may mediate cell-to-cell propagation of alpha-synuclein and in the formation of intracellular Lewy bodies^33^. Both FCGR2A and FCGR2B require binding to IgG-specific immune complexes to trigger their specific signaling cascades. The SomaScan panel used here does not distinguish between IgG isotypes. The identification and quantification of those isotypes in CSF could provide additional evidence to support or exclude FCGR2A and FCGR2B as being functionally linked to PD.

Out of the identified 68 causal proteins, only two reappear in the analysis of subcohorts: i.e., ARSA and CTSB. But while the former analysis is focused on casual effects on the whole patients’ group, the latter does not distinguish between cause and consequence and is subcohort-specific.

GBA and LRRK2 are enzymes involved in ceramide metabolism and lysosomal functions including the removal of aggregated alpha-synuclein. Genetic variants of the *GBA* and *LRRK2* genes are known risk factors for the development of PD and dementia associated with accumulation of Lewy bodies^34–36^. To better understand the differences between diseased and unaffected mutation carriers we compared their CSF proteomes (see Table 3).

Protein differences between *GBA* + patients and unaffected *GBA* + controls are related to brain dopaminergic neurons (DLK1), dopamine metabolism (CALCA, GCH1) and inflammation effector cells (IL17A). All these proteins have been previously linked to PD, though not specifically to *GBA*+. DLK1 is coupled to both tyrosine hydroxylase expression and neurotrophic signaling^37^. Significantly lower levels of DLK1 in *GBA* + patients suggest increased degeneration of dopaminergic neurons. GCH1 and CALCA are involved in nigrostriatal dopamine synthesis^38^, and dopamine release and metabolism^39,40^, respectively. We found both elevated in *GBA* + patients. *GCH1* variants contribute to the risk and earlier age-at-onset of PD^41^. IL17A was also augmented in the CSF of *GBA* + PD patients. It has been recently proposed that elevated IL17A plays a key role in neurodegenerative diseases^42^. Moreover, one of the inflammatory mechanisms proposed for neurodegeneration in PD involves Th17 cells^43^. Taking the above observations together, we hypothesize that *GBA* + induced loss of nigral dopaminergic neurons^44^ may trigger the neuroinflammatory events that lead to increased release of cytokines such as IL17A. IL17A in CSF maybe secreted by either Th17 brain resident cells and/or by Th17 cells infiltrating through the blood brain barrier^45^. Also, it cannot be excluded that IL17A could be also released by CD8+, lymphoid cells, NKTs and/or microglia.

Among the CSF proteins differentially expressed between *LRRK2* + patients and unaffected *LRRK2* + controls, several stood out for their relevance in PD: ARSA, SMPD1, CTSB and TENM4. ARSA (causal protein, see causal analysis) is a lysosomal chaperone that prevents alpha-synuclein aggregation, secretion and cell-to-cell propagation^46^. We found ARSA elevated in *LRRK2* + patients, which could be interpreted as a protection mechanism to prevent the formation of alpha-synuclein aggregates. CTSB (causal protein, see causal analysis) and SMPD1 play important roles in PD autophagy and lysosomal degradation processes^47^. *CTSB* and *SMPD1* genetic variants are known to be associated with PD risk^47^. In this study, higher CTSB and SMPD1 levels in *LRRK2* + patients indicate dysregulation of autophagy-endolysosomal pathway and potentially increased macroautophagy^48,49^. Brain TENM4 is involved in axon guidance and myelination^50^. In our study, it was significantly reduced in *LRRK2* + patients. Loss-of-function and missense variants in *TENM4* are associated with early onset PD and essential tremor, a potential risk factor for developing PD^51–60^.

Worldwide, ~90% of PD cases are idiopathic. Proteome differences between idiopathic patients and controls comprise not only markers of inflammation, but also of mitochondrial dysfunction/oxidative stress, iron metabolism and other pathological processes (see Table 3). The AK1 kinase is expressed by neurons and astrocytes^52^. At advanced PD stages, AK1 is downregulated in the substantia nigra probably due to mitochondrial dysfunction and dopaminergic neuronal death^52^. The observed elevation of CSF AK1 levels may be associated with PD progression stages, frontal cortex primary alteration or compensation of altered purine metabolism^52^. SHANK may be regulated by the mitochondrial kinase PINK1, for which variants are known to be causal for PD^53^. It has been reported that knockdown in neurons of *PINK* decreases PSD95 and SHANK1^53^. SHANK1 was decreased in idiopathic patients suggesting impaired synaptic plasticity. TXN promotes cell proliferation, protection against oxidative stress and anti-apoptotic functions in the brain, which makes it a good candidate for a neurodegeneration marker. Here we find TXN decreased in PD patients. Iron dysregulation is associated with oxidative stress and lipid peroxidation^54^. It has been proposed that LPO—heme peroxidase—in the substantia nigra is involved in neurodegeneration^55^. Lower LPO levels in idiopathic patients found here contrasts with previously reported elevated CSF LPO levels^55^. The high CSF levels of HAMP could help explaining this discrepancy. HAMP reduces iron accumulation and neuroinflammation by decreasing mitochondrial dysfunction, oxidative stress, and ultimately dopamine neuronal loss^56^. Moreover, HAMP overexpression—as seen here—promotes alpha-synuclein clearance through autophagy^57^. It has been also reported that dopamine and levodopa reduce LPO levels^55^. Given that the idiopathic patients recruited were drug-naïve early PD patients, dopamine levels in this subpopulation may have helped maintaining low levels of CSF LPO. The glucose metabolism enzyme GPI was elevated in idiopathic patients and may be protective. Its overexpression in dopaminergic neurons protects against alpha-synuclein-induced neurotoxicity^58^. As seen in *GBA* + patients, reduced DLK1 levels in idiopathic patients may suggest neurodegeneration. Lower levels of RAB31, a small GTPase involved in exosome biogenesis^59^ and potentially in alpha-synuclein spreading in PD^60^ were observed, too. RIPK2, a LRRK2 substrate, is lower in idiopathic patients, which matches the fact that LRRK2 deficiency leads to reduced activation of RIPK2^61^. The neurotrophic factor VEGFA is neuroprotective and has genetic variant associated with PD risk^62^. VIP enhances striatal plasticity and prevents dopaminergic cell loss in parkinsonian rats^63^. VEGFA and VIP at lower levels in idiopathic patients may reflect ongoing neurodegeneration.

Disease heterogeneity challenges the development of disease modifying therapies. In this study, we used proteomic data to stratify idiopathic patients into two clinically relevant endotypes.

The endotype robustness is confirmed by the high patient cluster stability (Fig. 3B) and the good performance of the endotype predictive model (see Fig. 3C). The fact that CSF phospho-tau levels sufficed to predict endotypes, suggests that targeted assessment of CSF proteins may be appropriate for idiopathic patient stratification in a clinical setting.

The lack of differences in the CSF levels of causal proteins, suggests that endotype molecular differences may be downstream from causal effects and affect the specific characteristics of PD phenotypes rather than PD risk. Pathway analysis of module proteins suggests inflammatory activity in endotype 2 (increased levels of proteins related to Th1, Th2, Th17 and macrophage activation pathways, as well as IL17 and other cytokines’ signaling pathways). In contrast, endotype 1 seems to have elevated levels of proteins related to synaptic connectivity and signaling, axonal guidance, and neurotransmission (Supplementary Fig. 1). Examples thereof could be glial fibrillary acidic protein, alpha-synuclein, microtubule associated protein tau, apolipoprotein E, 14-3-3 proteins (i.e., YWHAG, YWHAB, YWHAQ, YWHAZ). These results may reflect a higher neuroinflammatory activity for endotype 2, while activity for endotype 1 would be pointing to a compensation of the neurodegeneration process, as supported by the fact that endotype 1 shows less differences to controls while having enhanced levels of proteins related to neuronal function. Since endotypes showed differential progression as reflected by their ngPD-ProS, understanding their molecular differences might assist the design of personalized therapy approaches.

It is worth noting that, while in Tsukita et al.^17^ the ngPD-ProS perform similarly for both the idiopathic and the combined genetic subcohorts, here we see little overlap in the differentially expressed proteins between the idiopathic and each of the genetic subcohorts separately. This apparent discrepancy could just be reflecting a common CSF proteomic signature rising from the combination of genetic subcohorts even though each genetic subcohort may have different etiology. Supporting this hypothesis, when we compared the proteomes of the combined genetic subcohorts (*GBA*+ and *LRRK2*+) with their respective controls (data not shown) we found seven proteins shared with the 55 in the ngPD-ProS (i.e., DLK1, LPO, NEFH, RIPK2, SEMG2, VIP and LRFN2). These seven proteins intersected almost completely with the ten proteins that overlapped between the 55 proteins in the ngPD-ProS and the proteins differentially expressed between idiopathic patients and controls (i.e., DLK1, LPO, NEFH, RIPK2, SEMG2, VIP, CCL14, HAMP, RSPO4, and TXN). This similarity between the idiopathic subcohort and the combination of the genetic subcohorts could help explaining the apparent discrepancy mentioned above.

In summary, we: (i) identified causal proteins for PD, (ii) assessed CSF proteome differences in PD patients of genetic and idiopathic etiology, and (iii) stratified idiopathic patients into robust subtypes. Our findings not only contribute to the identification of new therapeutic targets but also to shaping personalized medicine in CNS neurodegeneration.

## Methods

The clinical data and samples used in this study were obtained from the PPMI (http://www.ppmi-info.org/data) on October 1, 2020. PPMI samples were collected under a standardized protocol over 33 centers and includes clinical and imaging data as well as plasma and CSF samples. Study protocol and manuals are available online (http://www.ppmi-info.org/study-design).

Separate subcohorts of patients with PD and their respective controls were enrolled following inclusion and exclusion criteria^64^. One subcohort is comprised of recently diagnosed, drug-naïve, idiopathic PD patients and healthy controls, while the second and third subcohorts are comprised of PD patients, carriers of a severe *GBA* or *LRRK2* mutation, either PD patients or unaffected controls. Parkinson’s disease patients from the genetic subcohorts had a longer disease duration, were partially under PD medication (*n* = 203), were over-represented for individuals of Ashkenazi Jewish descent and differed by sex distribution from the idiopathic PD patients (higher proportion of men among idiopathic). The study was approved by the Institutional Review Board at each site, and participants provided written informed consent.

Genetic testing was done by the centralized PPMI genetic testing core. Non-manifesting carriers received pre-testing and post-testing genetic counselling by phone from certified genetic counsellors at the University of Indiana or site-qualified personnel. The mutations considered severe in the *LRRK2* genetic testing battery included G2019S and R1441G. *GBA* genetic testing included five mutations considered severe: N370S (tested for all participants), and L483P, L444P, IVS2 + 1, and 84GG (tested for a subset of participants). Dual mutation carriers *(LRRK2* and *GBA)* were considered as *LRRK2* carriers for simplicity (*n* = 1).

Six patients were diagnosed as idiopathic PD at enrolment but were re-classified during follow-up (two patients were diagnosed as multiple system atrophy and four patients did not have a final diagnose but PD had been excluded). These patients were removed from the analysis. Four patients were initially diagnosed as genetic PD, but the diagnose changed to prodromal during follow up. These patients were considered as unaffected controls in their corresponding genetic subcohort (Five *LRRK2* + unaffected controls and one *GBA* + unaffected control).

One subject originally classified as healthy, but later shown to have an unclear health status, was removed from the analysis. Subjects recruited into the subcohort of idiopathic PD patients and healthy controls but identified as carriers of a severe *GBA* or *LRRK2* mutation, were moved to the corresponding genetic subcohort (*GBA n* = 15; *LRRK2 n* = 7).

The genetic screening also detected *GBA* mutations of unknown or moderate risk: A459P, E365K, T408M. Carriers of these mutations were removed from analysis (*n* = 38).

Finally, ten carriers of a mutation in *SCNA* (eight PD patients and two unaffected controls), were also removed due to lack of statistical power for analysis.

The original data set was comprised of 1190 samples out of which 32 samples were pools, which were discarded for this study, and as described above, additional six PD patients and one healthy control were removed due to change in diagnose, 38 subjects were removed due to non-severe *GBA* mutations, and 10 patients were removed for being carriers of a mutation in *SCNA*. Hence, the final data set used for analysis was comprised of 1103 proteomic samples divided into three subcohorts: no mutation (idiopathic PD patients and healthy controls with no severe mutation in *GBA* or *LRRK2*), *GBA* + (PD patient carriers of severe *GBA* mutations and unaffected controls carrying the same mutations) and *LRRK2* + (PD patients, carriers of severe *LRRK2* mutations and unaffected controls carrying the same mutations). The exact composition is summarized in Table 1.

**Table 1:**
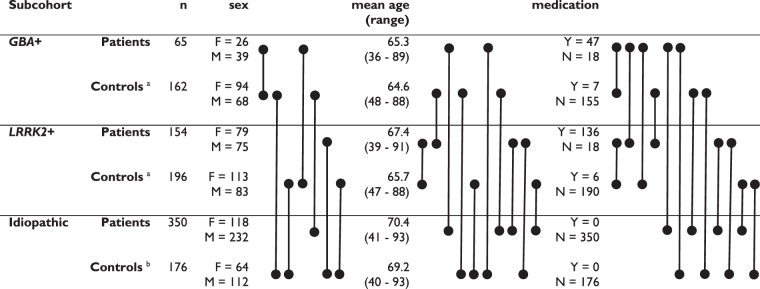
Parkinson’s disease subcohorts.

^a^“Unaffected controls” in the main text.

^b^“Healthy controls” in the main text.

Lines connecting dots highlight significant differences. Medication refers to use of levodopa or equivalent.

We make an explicit distinction between “healthy” and “unaffected” controls, because there is evidence of prodromal pathophysiology in *LRRK2* + and *GBA* + controls when compared to healthy controls that are non-mutation carriers^65^.

### Proteomics

Proteomic profiling was performed using SomaScan® in a platform version that is proprietary to Novartis and includes 4785 SOMAmers® (Slow Off-rate Modified Aptamers) targeting 4135 human proteins. SOMAmer levels were determined and standardized at SomaLogic Inc. (Boulder, US) including hybridization normalization (controls for variability in the readout of individual microarrays), plate scaling (accounts for plate-by-plate variation), median signal normalization (controls for total signal differences between individual samples) and calibration (removes the variation between assay runs within and across experiments).

Relative fluorescence units are transformed to log_2_ scale, normalized to the median separately by dilution level across all plates. Finally, the data set is adjusted for batch effects between plates using an empirical Bayes method as implemented in the R package *sva* v3.40.0^66^.

It should be noted that SomaScan does not discriminate protein isoforms or post-translational modifications. In turn it is to date the technology that allows quantifying the largest number of proteins in a single run, thus reasonably covering most biological pathways.

### Genetics

PPMI whole genome sequencing results were lifted over to hg19 coordinates. Biallelic SNPs on autosomes were extracted. Standard GWAS QC was applied at both individual and SNP level. 22 patients with outlying heterozygosity and 93 patients with high identity-by-descent were excluded after QC. 306031 SNPs were removed due to missing genotype and 35907596 SNPs removed due to minor allele count less than 20. Finally, 9743041 variants and 1264 subjects passed QC.

### pQTL calculation

Among the 1264 subjects who passed QC for genetics and the 1103 who passed QC for proteomics, 804 subjects overlapped. Protein expression values were ranked and inverse normal transformed. For pQTL calculation, each protein level was regressed with each independent genetic variant (SNP; Major Allele Frequency (MAF) > 0.05), adjusted for age, sex, subcohort, protein principal components 1–4 and genetic principal components 1–10 using R package *MatrixEQTL* v2.3^67^. Cis-pQTLs were defined as SNPs located inside the ±1 Mb region flanking the gene that encodes the given protein. A genome-wide threshold of *P* < 5 × 10^-8^ defined a significant cis-pQTL.

### Causal analysis

The two-sample mendelian randomization method implemented in the R package *TwoSampleMR* v0.5.6^68,69^ was applied to find causal proteins for PD in CSF. Although PPMI is a well-controlled study with genetic data, to avoid weak instrumental variable bias and take the advantage of a larger PD GWAS we relied on the meta-analysis from Nalls et al.^70^, which includes 17 datasets with PD cases ranging from 363 to 33674 individuals, and healthy controls ranging from 165 to 449056 individuals. Instrumental variables were selected for each SNP with MAF > 0.05, F-statistics larger than 10 and significant cis-pQTL *P* < 5 × 10^−8^. Shared SNPs in both cis-pQTL and PD GWAS were harmonized and then clumped using a linkage disequilibrium (LD) threshold of either *r*^*2*^ < 0.01 or *r*^*2*^ < 0.3. The more stringent LD threshold of *r*^2^ < 0.01 resulted in only one instrumental variable for most proteins, therefore the results we present are from the less stringent threshold of *r*^2^ < 0.3. The Wald ratio was used when only one instrument survived clumping, while the inverse variance weighted meta-analysis method was used when more than one instrumented SNP was available. Horizontal pleiotropy was tested using the R package *MRPRESSO* v1.0^71^.

Colocalization probability was calculated using the R package *coloc* v5.1.0^72^. Default priors of *p*_*1*_ = 10^−4^, *p*_*2*_ = 10^−4^, and *p*_*12*_ = 10^−5^ were used, where p_1_ is the prior probability of a SNP being associated with PD, p_2_ is the prior probability of a SNP being associated with CSF pQTL, and *p*_*12*_ is the prior probability of a SNP being associated with both PD and CSF pQTL. We considered *PPH*_*4*_ > 0.75 as strong evidence for colocalization. *PPH*_*4*_ is the posterior probability of one shared SNP being associated with both PD and CSF pQTL.

Whether mendelian randomization is enough to prove biological causality or not, is debatable. To facilitate readability, we will use the term “causal” as a simplification of “consistent with a potential causal effect”.

### Clinical variables

The clinical assessment battery is described on the PPMI website (http://www.ppmi-info.org). Parkinson’s disease status was assessed with the Unified Parkinson’s Disease Rating Scale in the revised version published by the Movement Disorder Society (MDS-UPDRS) scores 1, 2, and 3^73^. Cognitive testing comprised screening with the Montreal Cognitive Assessment (MoCA)^74^. High resolution xy-weighted 3 tesla MRI was available for 545 PD patients and 177 controls. Caudate, putamen and striatum thicknesses were calculated as the arithmetical mean between the values from the right and left brain hemispheres.

Cerebrospinal fluid was collected using standardized lumbar puncture procedures. Sample handling, shipment and storage were carried out as described in the PPMI biologics manual (http://ppmi-info.org). Besides the SomaScan analysis, data from immunoassay kits were also used for measuring CSF total amyloid-beta 1-42, total tau and phospho-tau (p-tau 181) and alpha-synuclein protein^75,76^. Briefly, for amyloid-beta 1-42 and total tau, standards, controls, and CSF samples were analyzed in duplicate with research-use-only Fujirebio-Innogenetics INNO-BIA AlzBio3 immunoassay kit–based reagents from Innogenetics Inc. The result was defined as the arithmetic mean of the calculated concentration of duplicates.

The amount of alpha-synuclein in CSF was analyzed by using a commercially available enzyme-linked immunosorbent assay kit from Covance. The concentration of alpha-synuclein was measured using standard alpha-synuclein curves (range, 6.1–1500 pg/mL using reconstituted stock) with 4-parameter curve fitting. The antibodies used in the kit do not cross react with beta-synuclein or gamma-synuclein.

Phospho-tau was measured with the Elecsys® assay run on the fully automated Roche Cobas® system.

Use of medications for PD was recorded at each visit after baseline assessment. For simplicity, we used this as a binary variable (medication present/absent).

### Protein risk scores

The PD protein risk scores used in this study were taken from Tsukita et al.^17^. They were defined as “non-genetic Parkinson’s Disease-associated Proteomic Score (ngPD-ProS).

### Statistical analysis

We first compared the protein profiles of PD patients with controls within each subcohort (idiopathic, *GBA*+ and *LRRK2*+ ). A linear regression model was applied using the Bioconductor R package *limma v3.48.3*^77^. The model included the following covariates: age, sex, study center and proteomic principal components 1–4. The genetic subcohorts also included levodopa treatment (yes/no) as a covariate in the model to exclude treatment effects. This was skipped for idiopathic patients as they were drug-naïve.

In addition, a linear model with an interaction term was tested. The interaction term was between the disease status (case /control) and the mutation status (no mutation/*GBA*+/*LRRK2*+). The covariates were the same as in the models above.

Comparisons of clinical variables between endotypes of idiopathic patients were performed using a chi-squared test (two-sided) for categorical variables or a generalized linear model adjusted for age and sex for quantitative variables. To test differences in age between endotypes a Mann Whitney U-test (two-sided) was used.

Predictive modeling for the idiopathic classes was performed using a partition tree with pruning as implemented in the R package *rpart v4.1.16*^78^. The model was defined on a training set (70% of the idiopathic patients) and tested on an independent test set (30% of the idiopathic patients). Ten-fold cross validation was used for pruning.

All *p* values were adjusted for multiple testing using false discovery rate (FDR). Our SomaScan v3 -based findings were indirectly validated by assessing whether SOMAmers measure the correct protein or not. For this purpose, sera proteomes from heart failure patients (*n* = 88) were assessed using both SomaScan v3 and Olink®, a high-throughput proteomics technology based on dual antibody target recognition. The values of 1329 SOMAmers overlapped with those from 804 unique Olink® assays (there is some SOMAmer redundancy in SomaScan technology). The Pearson’s correlation coefficient as well as the *p* value^79^ were calculated for each overlap (see Tables 2 and 3). It should be noted that a high correlation suggests that the SOMAmer is correctly measuring the correct protein. However, the opposite is not necessarily true. A poor correlation does not automatically discard the result, since the two platforms may target different isoforms or post-translationally modified proteins. Moreover, they could just be measuring semiquantitative protein abundances beyond the linear fraction of their dynamic range.

**Table 2:** Parkinson’s disease causal proteins from CSF.

Gene Symbol	SomaScan ID	Protein ID	Gene ID	Nr of IVs	Method	*β*	FDR	Colocalization PP.H4.abf	Horizontal Pleiotropy (*P* value)	Olink
GPNMB^a^	5080_131	Q14956	10457	11	IVW	0.14	1.17 × 10^−17^	0.94	0.65	OID05139 *r*^2^ = 0.762 *p* = 4.09 × 10^−17^
FCGR2B	3310_62	P31994	2213	17	IVW	0.07	5.11 × 10^−14^	0.96	0.49	OID00442 *r*^2^ = 0.837 *p* = 4.23 × 10^−23^
FCGR2A	3309_2	P12318	2212	18	IVW	0.07	2.70 × 10^−12^	0.95	0.26	OID01244 *r*^2^ = 0.392 *p* = 3.73 × 10−^4^
CTSB	8007_19	P07858	1508	12	IVW	−0.11	2.63 × 10^−10^	0.22	0.47	–
HLA-DQA2^b^	7757_5	P01906	3118	33	IVW	−0.14	1.43 × 10^−9^	0.03	0.00	–
CD38	11505_1	P28907	952	1	Wald ratio	−0.53	2.45 × 10^−9^	0.45	NA	OID00308 *r*^2^ = 0.003 *p* = 0.986
HP	3054_3	P00738	3240	19	IVW	0.06	2.01 × 10^−6^	0.01	0.60	–
LTF	2780_35	P02788	4057	23	IVW	0.05	2.16 × 10^−6^	0.04	0.65	–
HAVCR2	5134_52	Q8TDQ0	84868	13	IVW	−0.10	2.87 × 10^−5^	0.30	0.67	OID01410 *r*^2^ = 0.797 *p* = 1.52 × 10^−19^
BST1^b^	4535_50	Q10588	683	10	IVW	0.12	4.00 × 10^−5^	0.24	0.00	OID01436 *r*^2^ = 0.639 *p* = 7.99 × 10^−11^
CLEC3B	5701_81	P05452	7123	4	IVW	−0.16	1.02 × 10^−4^	0.29	0.97	–
HAPLN1	3196_6	P10915	1404	15	IVW	0.06	3.21 × 10^−4^	0.05	0.79	–
MANEA	8014_359	Q5SRI9	79694	17	IVW	0.05	3.21 × 10^−4^	0.10	0.94	–
NQO2	9754_33	P16083	4835	11	IVW	0.05	3.21 × 10^−4^	0.05	0.98	OID01173 *r*^2^ = 0.276 *p* = 0.018
ARSA^a^	3583_54	P15289	410	5	IVW	0.13	6.82 × 10^−4^	0.58	0.62	OID01479 *r*^2^ = 0.383 *p* = 5.41 × 10^−4^
LGALS3	3066_12	P17931	3958	8	IVW	0.07	7.11 × 10^−4^	0.02	0.50	OID00578 *r*^2^ = 0.584 *p* = 7.96 × 10^−9^
IL1RL1	4234_8	Q01638	9173	26	IVW	0.03	9.63 × 10^−4^	0.01	0.90	OID00634 *r*^2^ = 0.879 *p* = 4.45 × 10^−28^
PAM	5620_13	P19021	5066	12	IVW	0.09	9.63 × 10^−4^	0.08	0.47	OID01256 *r*^2^ = 0.496 *p* = 2.54 × 10^−6^
TPSAB1	9409_11	Q15661	64499 7177	8	IVW	−0.06	0.0011	0.15	0.80	OID00941 *r*^2^ = 0.803 *p* = 5.24 × 10^−20^
HSP90B1	6393_63	P14625	7184	22	IVW	0.04	0.0011	0.03	0.19	OID05159 *r*^2^ = 0.045 *p* = 0.772
GLCE	7808_5	O94923	26035	14	IVW	0.05	0.0015	0.10	0.96	–
MANSC4	9578_263	A6NHS7	100287284	18	IVW	0.05	0.0016	0.02	0.85	–
RABEPK	13599_15	Q7Z6M1	10244	10	IVW	−0.05	0.0021	0.10	0.86	–
ICAM1	4342_10	P05362	3383	8	IVW	−0.07	0.0021	0.32	0.74	OID01230 *r*^2^ = 0.099 *p* = 0.473
C4B^b,c^	2182_54	P0C0L4 P0C0L5	720 721	101	IVW	0.02	0.0021	0.00	0.00	–
LCT^b^	9017_58	P09848	3938	25	IVW	0.04	0.0021	0.00	0.00	–
SIRPB1	6247_9	O00241	10326	25	IVW	−0.03	0.0023	0.01	0.60	OID01430 *r*^2^ = 0.348 *p* = 0.002
C4A^b,c^	2182_54	P0C0L4 P0C0L5	720 721	101	IVW	0.02	0.0023	0.00	0.00	–
PCSK7	4459_68	Q16549	9159	18	IVW	−0.04	0.0028	0.02	0.98	–
IL9	5834_18	P15248	3578	12	IVW	0.06	0.0028	0.05	0.96	–
CLN5^a^	8874_53	O75503	1203	2	IVW	−0.15	0.0028	0.38	NA	–
AGT	3484_60	P01019	183	4	IVW	0.12	0.0039	0.06	0.97	–
CD274	5060_62	Q9NZQ7	29126	16	IVW	0.08	0.0042	0.02	0.46	OID00518 *r*^2^ = 0.120 *p* = 0.373
RBP7	14208_3	Q96R05	116362	3	IVW	0.20	0.0047	0.09	NA	–
PLA2G7	5015_15	Q13093	7941	14	IVW	0.04	0.0061	0.05	0.57	OID01283 *r*^2^ = 0.478 *p* = 6.71 × 10^−6^
EGF	5509_7	P01133	1950	3	IVW	−0.13	0.010	0.11	NA	OID00662 *r*^2^ = 0.762 *p* = 3.93 × 10^−17^
ASIP	5676_54	P42127	434	3	IVW	−0.13	0.010	0.17	NA	–
TPSB2	3403_1	P20231	64499	10	IVW	−0.05	0.011	0.15	0.27	–
ACP1	3858_5	P24666	52	15	IVW	0.04	0.011	0.04	0.88	–
RNASE3	5741_55	P12724	6037	6	IVW	0.09	0.011	0.01	0.26	OID01211 *r*^2^ = 0.920 *p* = 5.14 × 10^−35^
A4GALT	8759_29	Q9NPC4	53947	2	IVW	−0.21	0.012	0.11	NA	–
DSCAM	9175_48	O60469	1826	3	IVW	−0.30	0.015	0.19	NA	–
COLEC11	4430_44	Q9BWP8	78989	8	IVW	−0.05	0.016	0.03	0.61	–
SPOCK2	5491_12	Q92563	9806	8	IVW	0.08	0.017	0.03	0.98	–
VWA2	7128_9	Q5GFL6	340706	5	IVW	−0.11	0.018	0.04	0.76	–
RPN1	10490_3	P04843	6184	10	IVW	0.08	0.020	0.02	0.57	–
ADAMTS4	2809_25	O75173	9507	8	IVW	−0.06	0.020	0.19	0.36	–
PDCD1LG2	3004_67	Q9BQ51	80380	12	IVW	−0.09	0.020	0.04	0.07	OID00458 *r*^2^ = 0.740 *p* = 9.00 × 10^−16^
PRTN3	3514_49	P24158	5657	2	IVW	0.17	0.020	0.06	NA	OID00618 *r*^2^ = 0.779 *p* = 2.99 × 10^−18^
ADGRE2	4546_27	Q9UHX3	30817	10	IVW	−0.05	0.020	0.01	0.87	–
RNASE2	8394_56	P10153	6036	10	IVW	0.06	0.020	0.02	0.61	–
MPIG6B	7065_1	O95866	80739	18	IVW	−0.05	0.020	0.00	0.18	–
SIGLEC9	3007_7	Q9Y336	27180	19	IVW	0.03	0.020	0.02	0.99	OID00294 *r*^2^ = 0.311 *p* = 0.007
TAPBPL	6364_7	Q9BX59	55080	10	IVW	−0.03	0.020	0.03	0.50	–
LRP12^a^	7744_10	Q9Y561	29967	1	Wald ratio	0.73	0.023	0.66	NA	–
DNAJC30	7866_11	Q96LL9	84277	3	IVW	−0.09	0.024	0.03	NA	–
CCL15	3509_1	Q16663	6359	3	IVW	−0.08	0.024	0.12	NA	OID00629 *r*^2^ = 0.836 *p* = 6.47 × 10^−23^
VTN	13125_45	P04004	7448	22	IVW	−0.03	0.028	0.01	0.75	–
NUCB1	10451_11	Q02818	4924	3	IVW	−0.12	0.029	0.05	NA	–
TRH	5659_11	P20396	7200	4	IVW	−0.08	0.029	0.07	0.75	–
POSTN	6645_53	Q15063	10631	8	IVW	−0.07	0.034	0.02	0.76	–
PLXNB2^a^	10855_55	O15031	23654	36	IVW	−0.02	0.034	0.08	0.09	OID01218 rho = 0283 *p* = 0.015
IL18R1	14079_14	Q13478	8809	23	IVW	0.03	0.034	0.01	0.79	OID00517 *r*^2^ = 0.806 *p* = 2.64 × 10^−20^
IDUA	3169_70	P35475	3425	3	IVW	0.10	0.038	0.00	NA	OID00393 *r*^2^ = 0.743 *p* = 6.12 × 10^−16^
CFD	13678_169	P00746	1675	10	IVW	−0.05	0.039	0.01	0.58	–
GGH^a^	9370_69	Q92820	8836	4	IVW	0.08	0.040	0.02	0.49	–
FGFRL1	6237_11	Q8N441	53834	5	IVW	0.13	0.046	0.00	0.07	–
LMAN2L	8013_9	Q9H0V9	81562	3	IVW	−0.15	0.049	0.03	NA	–

Horizontal pleiotropy *p* value calculated with MRPRESSO.

By definition, all SOMAmers in Table 2 have a significant CSF cis pQTL according to Supplementary Table 5.

The “Olink” column represents the corresponding Olink ID, followed by the reversed Pearson’s correlation coefficient (*r*^2^) and the *p* value for the correlation.

^a^PD protein marker in Supplementary Table 3.

^b^Corrected by removing outliers by MRPRESSO.

^c^Has homolog detected by the same SOMAmer

**Table 3:** SOMAmers differentially expressed between Parkinson’s disease patient vs. controls, divided by subcohort.

Subcohort	Gene symbol	SomaScan ID	Protein ID	Gene ID	PD change direction	FDR	log2 FC	Olink	cis pQTL
*GBA*+	CALCA^39,40^^,^^83^	10494_48	P01258	796	+	0.022	0.071	OID01095 *r*^2^ = −0.046 *p* = 0.771	–
	CD2	7100_31	P06729	914	+	0.025	0.683	–	–
	DLK1^37^	6496_60	P80370	8788	‒	0.025	−0.184	OID00598 *r*^2^ = 0.831 *p* = 1.62 × 10^−22^	Yes
	GCH1^38,41^	11185_145	P30793	2643	+	0.025	0.118	–	–
	IL17A^42,43^	9170_24	Q16552	3605	+	0.025	0.089	OID00485 *r*^2^ = 0.109 *p* = 0.427	–
	SEMG2	6373_54	Q02383	6407	‒	0.022	−0.204	–	–
*LRRK2*+	ACP7	8011_96	Q6ZNF0	390928	+	0.043	0.644	–	Yes
	ARSA^46^	3583_54	P15289	410	+	0.023	0.339	OID01479 *r*^2^ = 0.383 *p* = 5.41 × 10^−4^	Yes
	CA10	13666_222	Q9NS85	56934	+	0.037	0.27	–	–
	^,^CTSB^25,29–31,47^	8007_19	P07858	1508	+	0.037 0.027^a^	0.222	–	Yes
	SIAE^16^	9263_57	Q9HAT2	54414	+	0.037	0.379	–	–
	SMPD1^47,48^	10818_36	P17405	6609	+	0.037	0.234	OID00309 *r*^2^ = 0.810 *p* = 1.32 × 10^−20^	–
	TENM4^50^^,^^84,85^	11365_17	Q6N022	26011	‒	0.037	−0.125	–	–
Idiopathic	AK1^52^	5012_67	P00568	203	+	0.049	0.107	–	Yes
	CCL14	2900_53	Q16627	6358	+	0.023	0.179	OID01292 *r*^2^ = 0.569 *p* = 2.32 × 10^−8^	Yes
	DLK1^37^	6496_60	P80370	8788	‒	0.032	−0.08	OID00598 *r*^2^ = 0.831 *p* = 1.62 × 10^−22^	Yes
	FRZB	13740_51	Q92765	2487	+	0.036	0.097	OID00312 *r*^2^ = 0.78 *p* = 2.27 × 10^−18^	Yes
	GPI^58^	4272_46	P06744	2821	+	0.006	0.152	–	–
	HAMP^56,57^	3504_58	P81172	57817	+	0.001	0.284	–	–
	LPO^55^	4801_13	P22079	4025	‒	1.5 × 10^-6^	−0.191	–	Yes
	LRRTM4	6572_10	Q86VH4	80059	‒	0.023	−0.073	–	–
	NETO1	5639_49	Q8TDF5	81832	‒	0.049	−0.08	–	–
	NFH	9900_36	P12036	4744	+	0.014	0.15	–	–
	PTHLH	2962_50	P12272	5744	‒	0.029	−0.144	–	Yes
	PTPRR	6361_49	Q15256	5801	‒	0.032	−0.066	–	–
	RAB31^60^	13597_20	Q13636	11031	‒	0.044	−0.077	–	–
	RELT	14112_40	Q969Z4	84957	‒	0.046	−0.051	OID01489 *r*^2^ = −0.001 *p* = 0.997	–
	RIPK2^61^	8993_151	O43353	8767	‒	4.6 × 10^−5^	−0.078	–	–
	ROBO3	5117_14	Q96MS0	64221	‒	0.049	−0.078	–	Yes
	RSPO4	8464_31	Q2I0M5	343637	‒	0.029	−0.076	–	Yes
	SEMG2	6373_54	Q02383	6407	‒	0.029	−0.084	–	–
	SHANK1^53^	13256_21	Q9Y566	50944	‒	0.023	−0.039	–	–
	SPINK9	8042_88	Q5DT21	643394	‒	0.040	−0.068	–	–
	TXN	10422_44	P10599	7295	‒	0.023	−0.057	–	–
	VEGFA^62^	2597_8	P15692	7422	‒	0.006	−0.073	OID00472 *r*^2^ = 0.751 *p* = 2.12 × 10^−16^	–
	VIP^63^	3522_57	P01282	7432	‒	0.023	−0.089	–	–

The change in PD represents (+) increased and (−) decreased in PD vs. controls, respectively. The reference numbers close to the gene symbol correspond to literature positions that link those genes with PD. The “Olink” column represents the corresponding Olink ID, followed by the reversed Pearson’s correlation coefficient (*r*^2^) and the *p* value for the correlation. The “pQTL” column indicates whether a significant CSF cis pQTL has been identified for the corresponding SOMAmer according to Supplementary Table 5.

^a^Interaction term.

### Cluster analysis

Network analysis of the CSF proteome of idiopathic patients was carried out using the R package *WGCNA* v1.70.3^80^. (WGCNA parameters: block-wise automatic module detection; soft threshold = 11; unsigned TOM, minimum module size = 500, Pearson correlation). Co-expressed proteins were grouped into modules of correlated proteins. Qiagen Ingenuity Pathway Analysis (IPA; July 2022 release) assisted unsupervised pathway analysis of proteins in modules.

Consensus clustering as implemented in the R package *ConsensusClusterPlus v1.56.0*^81^. used the SOMAmer modules to identify idiopathic patient subclasses. To avoid confounders being responsible for the differences between patient subclasses, network analysis was performed on the SOMAmer residuals of a linear regression on age, sex and study center.

Heatmaps were generated using the R package *Heatplus v3.0.0*^82^.

### Reporting summary

Further information on research design is available in the Nature Research Reporting Summary linked to this article.

## Supplementary information

Supplementary Materials

Reporting Summary

## Data Availability

The data used for this study is publicly available in the PPMI web page https://www.ppmi-info.org/access-data-specimens/download-data. The free access requires registration. The clinical data snapshot used here is kept under the tab “Archived PPMI data” » “Publication Associated Archives” » “2022-0001 Serrano-Fernandez: Parkinson’s Disease Proteogenomics (Version: 2022-05-18)”. The proteomic data is available under “Biospecimen” » “Proteomic Analysis” » “Project 151 Identification of proteins & protein networks & pQTL analysis in CSF x of 7 (Batch Corrected)” (7 files in total). The original adat files are also available under “Biospecimen” » “Proteomic Analysis” » “Project 151 Identification of proteins & protein networks & pQTL analysis in CSF - ADAT files”.
