# Supplementary material for: A proteogenomic view of Parkinson’s disease causality and heterogeneity
Source: NPJ Parkinsons Dis. 2023 Feb 11;9:24. doi: 10.1038/s41531-023-00461-9 (PMC9922273; doi:10.1038/s41531-023-00461-9)
Supplement: Supplementary file 1 — Supplementary Materials [file 41531_2023_461_MOESM1_ESM.pdf]

Supplementary material

Supplementary Table 1 Proteins with a significant cis-pQTL and their index SNPs

Gene symbol	RSID	CHR	POS	REF	ALT	ß	std.err	*P*-value
A4GALT	rs9611914	22	43206587	A	T	−0.24	0.04	5.92×10^{-10}
ABO	rs8176719	9	136132908	T	TC	1.18	0.03	2.93×10^{-190}
ACBD7	rs72774381	10	15127366	A	G	−0.43	0.06	3.56×10^{-12}
ACE	rs4335	17	61565025	G	A	−0.25	0.04	2.87×10^{-10}
ACP1	rs7595075	2	264019	C	A	1.15	0.03	8.70×10^{-179}
ACP5	rs78934187	19	11695690	A	G	0.60	0.04	5.81×10^{-43}
ACP6	rs2153463	1	147124310	T	G	1.08	0.03	4.95×10^{-185}
ACP7	rs1654245	19	39583160	G	A	−0.59	0.06	8.36×10^{-24}
ACYP2	rs3930909	2	54204922	C	T	−0.33	0.06	9.55×10^{-9}
ADA2	rs2231495	22	17669306	T	C	−1.00	0.03	8.47×10^{-129}
ADAM12	rs10794080	10	128053754	T	C	0.37	0.04	3.61×10^{-22}
ADAM9	rs62504410	8	38822313	T	C	0.14	0.02	6.63×10^{-9}
ADAMTS1	rs56251528	21	28210854	C	T	−0.61	0.09	7.28×10^{-11}
ADAMTS13	rs28647808	9	136305530	C	G	−0.46	0.07	1.19×10^{-10}
ADAMTS4	rs4233367	1	161163037	T	C	0.66	0.04	1.76×10^{-60}
ADAMTS5	rs2830580	21	28291149	C	T	−1.00	0.04	1.50×10^{-104}
ADAMTSL1	rs1832638	9	18494190	T	C	0.29	0.03	3.80×10^{-18}
ADGRE2	rs7256892	19	14878003	C	T	−0.55	0.04	4.17×10^{-33}
ADGRF5	rs475991	6	46822349	A	G	0.24	0.04	1.20×10^{-8}
ADH1B	rs1229984	4	100239319	T	C	0.37	0.06	1.25×10^{-9}
ADM	rs10743132	11	10266998	A	G	0.20	0.03	2.10×10^{-11}
AGRP	rs4360931	16	67471926	C	G	0.65	0.07	4.44×10^{-19}
AGT	rs12028675	1	230832275	A	G	−0.36	0.04	1.97×10^{-23}
AHSG	rs4917	3	186337713	T	C	−1.27	0.03	2.87×10^{-216}
AK1	rs73602391	9	130661356	G	A	−0.49	0.08	9.71×10^{-10}
AKR1A1	rs7520156	1	45912262	A	T	−1.57	0.07	4.15×10^{-83}
AKR1B1	rs113764574	7	134207748	G	A	−0.20	0.04	2.54×10^{-8}
AKR1C1	rs11594322	10	4956083	A	C	−0.44	0.05	1.92×10^{-16}
AKR7A2	rs11800204	1	19576096	T	C	−0.42	0.05	1.67×10^{-18}
AMIGO2	rs58366817	12	47934532	T	C	−0.27	0.04	3.32×10^{-10}
AMY2B	rs17014910	1	104104990	C	G	0.28	0.04	2.12×10^{-11}

ANG	rs12433905	14	21158303	G	A	0.62	0.06	1.17×10^{-20}
ANGPTL1	rs61823772	1	178925881	C	G	−0.33	0.04	2.22×10^{-16}
ANGPTL4	rs34099346	19	8503345	G	C	0.33	0.06	3.02×10^{-9}
ANGPTL7	rs28729193	1	11180151	T	C	−0.20	0.04	1.59×10^{-8}
ANXA2	rs12900365	15	60644463	T	A	0.20	0.03	2.62×10^{-9}
ANXA5	rs71606236	4	122580725	T	C	−0.33	0.04	3.16×10^{-14}
AOC1	rs62492368	7	150537635	G	A	0.92	0.04	3.68×10^{-94}
APCDD1	rs3748415	18	10471732	G	A	−0.43	0.07	1.27×10^{-10}
APOC2	rs429358	19	45411941	T	C	0.89	0.06	3.56×10^{-39}
APOE	rs429358	19	45411941	T	C	0.82	0.05	2.49×10^{-45}
APOH	rs8178824	17	64224775	C	T	−1.63	0.08	1.13×10^{-78}
APOL1	rs10854688	22	36653854	C	T	0.20	0.03	4.54×10^{-9}
ARHGAP1	rs79920646	11	46934260	C	G	−0.32	0.05	6.83×10^{-11}
ARHGAP25	rs10048745	2	68962137	G	A	−0.40	0.04	3.25×10^{-23}
ARSA	rs6151419	22	51064915	G	A	−0.51	0.05	2.01×10^{-22}
ARSB	rs13159135	5	78196689	G	C	−1.02	0.03	1.30×10^{-127}
ARSK	rs9314144	5	94797432	T	C	−0.60	0.08	2.55×10^{-12}
ART3	rs12509988	4	76989108	A	G	−0.65	0.05	9.32×10^{-37}
ASAH2	rs202183815	10	51994615	C	T	−0.79	0.04	6.27×10^{-62}
ASIP	rs62209647	20	32505658	G	C	0.62	0.08	2.87×10^{-13}
ASL	rs77627477	7	66166078	CTG	C	−0.34	0.04	6.31×10^{-17}
ASPN	rs10120980	9	95020914	A	G	−0.26	0.04	3.79×10^{-11}
ATF6B	rs805262	6	31628733	C	T	0.17	0.03	4.17×10^{-8}
ATP1B2	rs1642764	17	7557834	C	T	−0.20	0.03	7.29×10^{-10}
B3GAT3	rs10897288	11	62386472	A	T	0.37	0.03	3.67×10^{-26}
B3GLCT	rs11147458	13	31823239	A	G	−0.41	0.05	4.93×10^{-16}
B3GNT8	rs284663	19	41932612	C	T	0.68	0.03	2.70×10^{-70}
B4GALNT1	rs34919374	12	58053464	G	A	0.35	0.04	2.46×10^{-14}
B4GALT1	rs4879664	9	33108246	T	G	−0.19	0.03	1.77×10^{-9}
B4GALT2	rs3216995	1	44443874	T	TGAC	−0.56	0.06	3.06×10^{-22}
B4GALT6	rs16962266	18	29232293	T	C	−0.56	0.04	7.35×10^{-33}
BCAM	rs1135062	19	45322744	A	G	0.26	0.03	1.02×10^{-13}
BCAN	rs2365715	1	156615114	A	G	−0.51	0.04	2.46×10^{-38}

BCHE	rs3908218	3	165462643	A	G	−0.27	0.04	9.99×10^{-12}
BOC	rs3856719	3	112996541	T	C	0.40	0.05	4.30×10^{-16}
BPI	rs1341024	20	36932676	G	C	0.20	0.03	3.92×10^{-15}
BPIFB1	rs11699009	20	31688241	T	C	−0.43	0.05	4.38×10^{-20}
BRPF1	rs2269112	3	9788168	C	T	0.61	0.05	1.42×10^{-27}
BST1	rs4263397	4	15739390	T	G	−1.12	0.04	2.91×10^{-141}
BTD	rs6784108	3	15739777	C	A	−0.38	0.04	9.03×10^{-20}
BTN2A1	rs2237235	6	26391395	A	G	0.50	0.03	7.10×10^{-42}
BTN3A1	rs9379851	6	26354780	A	C	−1.14	0.05	3.43×10^{-84}
BTNL9	rs34492237	5	180295971	C	A	0.27	0.04	1.04×10^{-9}
C1QC	rs12058824	1	22963050	G	A	0.67	0.08	3.52×10^{-16}
C1QL1	rs4793169	17	43152877	T	C	−0.30	0.03	3.13×10^{-17}
C1QL4	rs7972869	12	49731167	A	G	0.36	0.06	3.88×10^{-10}
C1QTNF3	rs7712366	5	34038537	A	G	0.56	0.04	4.57×10^{-34}
C1QTNF5	rs2248863	11	119207341	G	A	0.52	0.07	3.17×10^{-14}
C1S	rs7962629	12	7166770	A	G	−0.37	0.06	6.63×10^{-11}
C2	rs116198852	6	31393014	C	T	−0.52	0.07	5.43×10^{-13}
C4A	rs2280774	6	31928691	G	A	−0.95	0.04	9.57×10^{-105}
C4B	rs2280774	6	31928691	G	A	−0.95	0.04	9.57×10^{-105}
C7	rs147512353	5	40971249	T	C	−0.32	0.05	2.40×10^{-10}
CA4	rs923038	17	58220132	T	G	−0.63	0.08	2.09×10^{-15}
CA6	rs3765963	1	9034598	A	G	0.32	0.04	1.55×10^{-13}
CA9	rs2071676	9	35674053	G	A	0.28	0.04	2.66×10^{-10}
CABLES2	rs2427312	20	60970591	C	T	−0.32	0.04	1.70×10^{-15}
CALCOCO2	rs550510	17	46926615	G	A	−0.30	0.04	3.91×10^{-14}
CAPG	rs4446071	2	85543064	T	C	−0.42	0.05	1.27×10^{-18}
CAPSL	rs6865274	5	35923829	A	G	−0.39	0.07	1.28×10^{-8}
CAT	rs3781709	11	34504711	T	C	−0.32	0.05	5.31×10^{-10}
CBR1	rs1005696	21	37443480	T	G	−0.30	0.04	8.01×10^{-12}
CBR3	rs1056892	21	37518706	G	A	−0.60	0.03	4.04×10^{-60}
CCDC50	rs56134151	3	191045679	C	T	−0.20	0.04	9.99×10^{-9}
CCL14	rs7222922	17	34335694	C	T	−0.92	0.05	1.58×10^{-63}

CCL15	rs41508645	17	34329044	T	G	1.13	0.06	7.86×10^{-58}
CCL16	rs112689088	17	34307457	T	C	−1.30	0.05	5.62×10^{-95}
CCL17	rs223896	16	57443146	G	A	−0.37	0.04	3.37×10^{-16}
CCL18	rs2015086	17	34391617	A	G	0.90	0.06	1.63×10^{-47}
CCL23	rs7208990	17	34329475	C	G	−0.79	0.08	4.05×10^{-23}
CCL25	rs62124694	19	8126830	A	C	0.27	0.05	2.99×10^{-8}
CCL3L1	rs56683451	17	34374696	T	C	0.45	0.07	3.29×10^{-11}
CCL7	rs3138036	17	32647544	A	G	−0.50	0.06	3.47×10^{-14}
CCL8	rs3138036	17	32647544	A	G	−1.25	0.05	2.78×10^{-106}
CCN3	rs2469990	8	120344567	A	G	−0.39	0.05	2.74×10^{-14}
CCN4	rs2739063	8	133948522	A	G	−1.03	0.03	2.63×10^{-132}
CCN5	rs11086936	20	43331807	C	G	0.44	0.03	6.73×10^{-33}
CD109	rs10223501	6	74419424	C	T	0.41	0.04	5.84×10^{-25}
CD14	rs12517200	5	140019921	A	G	0.34	0.03	2.33×10^{-21}
CD177	rs78530667	19	43900697	T	C	−0.74	0.07	2.59×10^{-23}
CD209	rs4804774	19	7772173	G	C	−0.57	0.05	1.94×10^{-29}
CD274	rs7048841	9	5460801	T	C	−0.53	0.03	2.48×10^{-66}
CD300A	rs2272111	17	72469966	G	A	−0.58	0.04	3.80×10^{-49}
CD300C	rs2670826	17	72462466	C	T	−0.28	0.04	4.05×10^{-14}
CD300E	rs581157	17	72613589	T	G	−0.67	0.04	5.52×10^{-56}
CD33	rs3865444	19	51727962	C	A	−1.03	0.03	2.15×10^{-154}
CD38	rs6836946	4	15813607	T	G	0.31	0.05	5.73×10^{-10}
CD48	rs12124234	1	160675269	G	C	0.28	0.05	5.15×10^{-9}
CD55	rs116059019	1	207572442	G	A	0.56	0.05	6.98×10^{-26}
CD59	rs3181269	11	33755956	C	T	−0.23	0.03	4.57×10^{-15}
CD72	rs7026779	9	35581458	A	G	0.71	0.04	1.14×10^{-48}
CD83	rs11756936	6	14110109	G	C	0.27	0.03	2.53×10^{-15}
CD84	rs796499427	1	160574660	AAC	A	−0.50	0.06	1.34×10^{-15}
CD8A	rs3020726	2	87016506	A	G	0.67	0.06	2.42×10^{-26}
CD93	rs11697283	20	23108635	A	G	0.25	0.04	1.50×10^{-8}
CDCP1	rs2276862	3	45187785	C	G	−0.43	0.05	1.10×10^{-16}
CDH11	rs1124695	16	65037770	C	G	−1.26	0.04	5.97×10^{-154}
CDNF	rs72772427	10	14911640	C	T	0.65	0.04	4.93×10^{-57}

CDON	rs35088114	11	125842801	G	GGCA	−0.53	0.06	1.63×10^{-18}
CEACAM19	rs429358	19	45411941	T	C	0.46	0.05	4.05×10^{-17}
CEL	rs668809	9	135936678	A	G	1.28	0.10	8.17×10^{-37}
CER1	rs2131882	9	14716849	T	A	0.89	0.06	6.77×10^{-44}
CFB	rs9296004	6	31933977	A	C	−0.47	0.07	2.41×10^{-10}
CFD	rs71335276	19	859368	G	T	0.93	0.05	5.16×10^{-74}
CFH	rs35617250	1	196679682	C	T	0.40	0.05	1.35×10^{-13}
CFHR1	rs71631867	1	196814850	T	A	−0.86	0.04	1.01×10^{-81}
CFHR4	rs200880049	1	196790437	T	A	−0.17	0.03	2.38×10^{-11}
CFHR5	rs1694459	1	196815786	C	T	0.33	0.04	1.04×10^{-13}
CFI	rs7439493	4	110656730	G	A	0.21	0.04	2.68×10^{-8}
CGB3	rs78248023	19	49515171	C	A	0.97	0.06	2.72×10^{-50}
CGB7	rs78248023	19	49515171	C	A	0.97	0.06	2.72×10^{-50}
CHGA	rs729940	14	93399101	C	T	−0.27	0.04	4.92×10^{-12}
CHI3L1	rs946259	1	203152177	C	T	−1.07	0.03	2.09×10^{-183}
CHI3L2	rs11556868	1	111778325	C	T	1.42	0.05	8.36×10^{-127}
CHIT1	rs2486063	1	203173094	A	G	−1.04	0.05	7.77×10^{-82}
CHRD	rs73053799	3	184111595	C	T	0.36	0.05	8.46×10^{-13}
CHRDL2	rs7104802	11	74386800	G	T	0.35	0.03	7.22×10^{-27}
CHST12	rs3735099	7	2472429	C	A	0.73	0.06	5.51×10^{-32}
CHST9	rs12968277	18	24637729	G	C	0.91	0.03	4.71×10^{-119}
CLEC11A	rs11666008	19	51226590	C	G	−0.35	0.05	2.93×10^{-11}
CLEC12A	rs607567	12	10131684	T	G	−1.12	0.03	3.05×10^{-179}
CLEC2L	rs12672312	7	139230464	C	T	0.16	0.02	5.49×10^{-11}
CLEC3B	rs4683019	3	45040682	T	C	−0.33	0.03	4.61×10^{-23}
CLEC4C	rs10845821	12	7900184	C	T	0.56	0.04	4.37×10^{-35}
CLEC4G	rs115293707	19	7794609	G	C	1.39	0.07	1.07×10^{-73}
CLEC7A	rs16910526	12	10271087	A	C	−1.48	0.07	2.48×10^{-82}
CLIC5	rs76820035	6	45919515	G	A	0.78	0.08	8.69×10^{-21}
CLN5	rs2794618	13	77584224	G	A	−0.71	0.07	1.32×10^{-21}
CLPS	rs12193764	6	35742556	C	G	0.48	0.05	3.24×10^{-21}
CNDP1	rs4329999	18	72228269	G	A	−0.48	0.04	1.20×10^{-28}
CNTN2	rs41264869	1	205030862	C	T	−0.68	0.05	3.66×10^{-32}

CNTN4	rs163352	3	3098041	G	C	−0.18	0.02	8.68×10^{-13}
CNTN5	rs72998295	11	99093036	T	C	−0.25	0.04	4.89×10^{-9}
COCH	rs66816140	14	31239287	AC	A	0.67	0.04	2.71×10^{-47}
COL11A2	rs17847933	6	33165780	G	C	0.74	0.08	3.51×10^{-20}
COL15A1	rs7025094	9	101667626	G	A	0.31	0.04	1.26×10^{-12}
COL18A1	rs12483377	21	46931109	G	A	−0.42	0.06	1.01×10^{-10}
COL23A1	rs4976753	5	177681651	C	T	−0.81	0.09	1.36×10^{-17}
COLEC11	rs11123637	2	3654190	T	C	−1.06	0.03	3.63×10^{-140}
CPA4	rs34587586	7	129938598	G	T	−1.14	0.03	2.48×10^{-176}
CPB1	rs13079446	3	148509614	T	A	−0.84	0.06	6.26×10^{-38}
CPB2	rs9567615	13	46651080	A	C	0.30	0.04	2.91×10^{-12}
CPE	rs72703629	4	166346061	T	C	0.27	0.03	2.73×10^{-15}
CPM	rs12372220	12	69371471	C	T	−0.77	0.05	1.21×10^{-55}
CPN2	rs6809081	3	194060475	T	C	−0.34	0.04	8.78×10^{-15}
CPXM1	rs11697820	20	2781441	C	T	0.91	0.04	4.39×10^{-101}
CPZ	rs13121547	4	8601592	G	T	−0.51	0.04	1.27×10^{-29}
CREB3L4	rs140257179	1	153963995	A	AAG	0.38	0.04	2.57×10^{-19}
CRELD1	rs6762702	3	10055789	G	A	−0.62	0.04	6.18×10^{-58}
CRHBP	rs6871700	5	76209399	A	G	−0.28	0.04	3.10×10^{-10}
CRIM1	rs10189344	2	36778887	A	C	−0.22	0.03	9.03×10^{-12}
CRISP2	rs10948527	6	49746832	C	T	−0.44	0.05	1.07×10^{-18}
CRISPLD2	rs1466716	16	84870034	T	C	0.51	0.06	8.58×10^{-16}
CRLF1	rs3761031	19	18721451	G	A	0.39	0.04	2.07×10^{-22}
CROT	rs7776867	7	87014355	C	T	−0.55	0.08	1.09×10^{-11}
CRTAC1	rs10883027	10	99798234	G	C	0.16	0.02	3.21×10^{-14}
CRYGD	rs55770589	2	208985427	T	C	0.32	0.03	2.15×10^{-24}
CRYZ	rs3819946	1	75175886	T	C	1.02	0.04	2.91×10^{-101}
CSGALNACT2	rs2435381	10	43678796	C	T	−0.98	0.04	6.93×10^{-118}
CST1	rs113896553	20	23731589	G	C	1.10	0.03	1.24×10^{-143}
CST2	rs113896553	20	23731589	G	C	1.09	0.03	1.92×10^{-148}
CST3	rs2983644	20	23592966	C	A	0.38	0.03	3.93×10^{-30}
CST4	rs7264810	20	23673625	C	A	−0.54	0.07	1.19×10^{-13}

CST5	rs4239743	20	23859017	A	C	0.69	0.04	3.04×10^{-59}
CST6	rs1131544	11	65779590	C	T	1.18	0.04	1.02×10^{-126}
CST7	rs148851012	20	24973964	ATTTGATAAAGTTTATTAAGT	A	0.85	0.07	2.74×10^{-32}
CTHRC1	rs10093100	8	104368191	T	C	0.41	0.04	3.27×10^{-19}
CTNNA2	rs72917277	2	79549662	A	G	−0.41	0.06	1.26×10^{-10}
CTRB1	rs113366803	16	75299391	A	G	0.45	0.08	3.42×10^{-8}
CTRB2	rs889515	16	75251299	T	C	−1.00	0.05	1.35×10^{-65}
CTSB	rs28577034	8	11708715	A	T	−0.68	0.04	1.76×10^{-50}
CTSC	rs11600158	11	88070914	A	G	0.89	0.07	3.39×10^{-37}
CTSF	rs1127894	11	66335548	A	G	0.26	0.04	7.79×10^{-12}
CTSH	rs2289702	15	79237293	C	T	−1.49	0.06	9.07×10^{-90}
CTSK	rs55890313	1	150640078	T	A	−0.47	0.07	5.00×10^{-12}
CTSO	rs138798877	4	156936516	ATATT	A	−0.46	0.08	3.91×10^{-8}
CTSS	rs41271951	1	150737220	A	G	−1.26	0.09	1.04×10^{-41}
CTSZ	rs163787	20	57574463	A	G	0.40	0.05	3.58×10^{-14}
CXCL1	rs7438101	4	74728327	T	C	−0.33	0.05	9.90×10^{-10}
CXCL6	rs62312418	4	74701512	G	A	0.35	0.04	5.70×10^{-16}
CYB5D2	rs35103069	17	4108063	G	C	−0.21	0.03	2.44×10^{-9}
CYTL1	rs6446312	4	5019932	C	G	−0.69	0.05	9.77×10^{-39}
DEFB1	rs2978857	8	6741305	T	G	−1.18	0.03	3.47×10^{-157}
DKK2	rs17037069	4	107838076	A	G	−0.62	0.07	4.69×10^{-16}
DLK1	rs1058009	14	101200860	G	A	−0.62	0.09	8.54×10^{-12}
DLL1	rs9459968	6	170497591	C	T	−0.32	0.04	5.38×10^{-18}
DNAJC10	rs746705411	2	183612210	CTT	C	0.18	0.03	2.00×10^{-9}
DNAJC30	rs10479665	7	73108924	A	G	−0.88	0.10	4.14×10^{-18}
DNER	rs67033065	2	230532197	ATCCT	A	−0.19	0.02	3.40×10^{-17}
DPP7	rs6560658	9	140004229	C	G	0.82	0.05	4.12×10^{-57}
DPT	rs12048750	1	168922582	G	A	0.44	0.04	6.08×10^{-22}
DRAXIN	rs1555079	1	11760782	C	T	−0.71	0.04	6.37×10^{-72}
DSC2	rs1789064	18	28674402	A	T	−0.49	0.04	8.70×10^{-35}

DSC3	rs1370450	18	28741821	G	A	−0.23	0.04	7.35×10^{-9}
DSCAM	rs401936	21	41449867	C	T	0.27	0.02	1.71×10^{-26}
DSG2	rs9304098	18	29083630	G	T	−0.45	0.04	6.00×10^{-24}
EBI3	rs4740	19	4236996	G	A	−1.18	0.03	1.95×10^{-179}
ECH1	rs2229259	19	39307103	C	T	0.58	0.06	2.98×10^{-18}
ECM1	rs9326002	1	150495106	A	G	−0.42	0.03	2.03×10^{-51}
EDAR	rs260689	2	109579110	G	A	0.36	0.06	2.60×10^{-8}
EDIL3	rs13160212	5	83219474	T	C	0.42	0.05	2.60×10^{-19}
EFEMP1	rs1430192	2	56117040	T	C	0.39	0.03	1.14×10^{-32}
EFNA5	rs7709385	5	107047072	G	T	−0.18	0.03	1.23×10^{-8}
EGF	rs4698801	4	110882476	C	A	0.38	0.04	6.48×10^{-24}
EGFLAM	rs12516477	5	38245951	T	C	0.84	0.08	1.81×10^{-26}
EGFR	rs151057105	7	54944920	C	T	−0.66	0.04	4.80×10^{-43}
EHF	rs286924	11	34642728	A	T	−0.59	0.09	1.81×10^{-10}
EMILIN3	rs6102384	20	39997745	T	C	0.41	0.07	1.05×10^{-8}
ENDOU	rs7976708	12	48116255	A	G	−0.58	0.05	1.03×10^{-26}
ENPP5	rs62400861	6	46100229	C	G	−1.52	0.04	3.85×10^{-173}
ENPP7	rs36069406	17	77696496	GC	G	0.80	0.04	4.56×10^{-78}
ENTPD3	rs2276868	3	40498845	C	T	0.31	0.04	2.08×10^{-13}
ENTPD6	rs3859660	20	25915180	A	T	0.68	0.07	1.47×10^{-20}
EPDR1	rs2044831	7	37988589	C	T	−0.35	0.05	7.07×10^{-13}
EPHA1	rs4725617	7	143097100	A	G	1.44	0.08	4.17×10^{-66}
EPHA10	rs12049522	1	38245517	T	G	−0.14	0.02	1.40×10^{-8}
EPHA2	rs28452540	1	16423834	A	G	−0.23	0.04	2.11×10^{-9}
EPHA5	rs10434242	4	66518523	A	G	0.14	0.02	1.65×10^{-9}
EPO	rs114129687	7	100290926	T	G	−0.39	0.04	2.79×10^{-20}
ERAP1	rs26653	5	96139250	C	G	−0.95	0.03	7.78×10^{-117}
ERAP2	rs2927608	5	96252432	G	A	1.23	0.03	1.53×10^{-225}
ERBB4	rs12987104	2	213302799	A	G	−0.27	0.04	6.13×10^{-11}
ERLEC1	rs17039525	2	53955513	A	C	−0.14	0.02	1.04×10^{-9}
ERLIN1	rs2862954	10	101912064	T	C	0.36	0.04	4.48×10^{-15}
ERO1B	rs2477599	1	236413230	T	A	−1.01	0.03	4.86×10^{-145}
ESD	rs1216970	13	47374012	C	G	−1.29	0.06	7.03×10^{-76}

ESM1	rs78414307	5	54434781	TA	T	−0.38	0.05	5.97×10^{-12}
EYS	rs9342464	6	66005888	C	T	0.22	0.04	4.08×10^{-8}
F11	rs6824705	4	187213889	C	T	0.30	0.04	4.64×10^{-14}
F13B	rs35258876	1	197151626	C	CA	−0.25	0.04	6.33×10^{-11}
F5	rs72708013	1	169481731	T	G	−0.87	0.09	1.13×10^{-20}
FABP1	rs2241883	2	88424066	T	C	−0.24	0.04	1.05×10^{-10}
FABP3	rs879491795	1	31676195	AG	A	0.24	0.04	5.61×10^{-9}
FABP6	rs10063628	5	159631958	C	G	−0.61	0.05	8.47×10^{-35}
FAH	rs34239630	15	80441888	C	T	−0.35	0.05	1.47×10^{-11}
FAIM	rs641320	3	138347957	G	A	1.47	0.08	2.43×10^{-69}
FAM177A1	rs79698726	14	35522350	A	C	−0.22	0.03	6.18×10^{-12}
FAM20A	rs12602247	17	66685034	C	G	0.30	0.04	6.23×10^{-11}
FAM234B	rs200946929	12	13201461	TCTG TTAT CC	T	0.18	0.03	1.74×10^{-8}
FAM3B	rs66817580	21	42689637	G	T	1.42	0.04	1.50×10^{-147}
FAM3D	rs3749290	3	58652292	G	T	−0.67	0.06	4.38×10^{-28}
FAS	rs6586163	10	90752018	A	C	0.90	0.03	6.05×10^{-121}
FBP1	rs28369691	9	97384265	C	T	0.68	0.08	8.56×10^{-17}
FBP2	rs10761342	9	97346079	C	T	0.28	0.05	2.90×10^{-9}
FCER1A	rs6703348	1	159291683	C	G	0.61	0.04	1.44×10^{-38}
FCER2	rs12980031	19	7764436	G	T	−1.08	0.03	2.97×10^{-141}
FCGR2A	rs1801274	1	161479745	A	G	1.23	0.02	5.70×10^{-262}
FCGR2B	rs1801274	1	161479745	A	G	1.05	0.03	2.26×10^{-137}
FCGR3B	rs2487452	1	161530869	G	A	0.47	0.05	1.38×10^{-21}
FCN2	rs7037264	9	137775212	G	A	−0.65	0.04	1.97×10^{-53}
FCRL6	rs12088352	1	159784539	G	A	−0.37	0.04	2.95×10^{-17}
FGF17	rs77473749	8	21877382	C	G	1.19	0.08	3.37×10^{-46}
FGF19	rs9667380	11	69611959	C	T	0.33	0.04	2.83×10^{-17}
FGFBP3	rs10881987	10	93641869	A	G	0.40	0.04	1.61×10^{-21}
FGFRL1	rs4647930	4	1018705	C	A	0.35	0.04	1.44×10^{-17}
FKBP14	rs28619411	7	30096002	A	T	0.37	0.03	3.18×10^{-39}
FKBP7	rs2886712	2	179311529	G	A	0.64	0.03	5.55×10^{-72}
FLRT1	rs588177	11	64024056	C	A	0.18	0.03	2.92×10^{-8}

FLRT2	rs10498583	14	85742934	G	A	−0.17	0.03	1.85×10^{-8}
FLRT3	rs150296190	20	14822666	T	TTTTG	−0.21	0.03	1.38×10^{-14}
FMOD	rs28583560	1	203315331	G	A	−0.71	0.09	8.67×10^{-15}
FOLH1	rs56287037	11	49106344	G	A	1.49	0.10	1.33×10^{-44}
FREM2	rs34692381	13	39331723	C	T	−0.63	0.04	1.64×10^{-55}
FRZB	rs288326	2	183703336	G	A	0.79	0.06	5.16×10^{-40}
FSTL1	rs66685284	3	120172964	AAC	A	0.30	0.05	3.58×10^{-11}
FSTL3	rs71333301	19	676904	CGA	C	−0.24	0.04	2.87×10^{-11}
FSTL5	rs2314103	4	162334838	T	C	−0.58	0.06	9.24×10^{-20}
FUT10	rs16880849	8	33246538	A	G	0.53	0.03	2.24×10^{-54}
FUT5	rs72989070	19	5895358	C	T	0.91	0.03	5.33×10^{-115}
FUT8	rs7140341	14	66070417	C	G	0.29	0.04	4.46×10^{-13}
GALNT10	rs1106324	5	153580836	A	G	0.24	0.04	1.10×10^{-8}
GALNT16	rs7143324	14	69755470	A	G	−0.32	0.03	7.71×10^{-19}
GCA	rs17783344	2	163208893	T	G	−0.40	0.05	7.10×10^{-18}
GDF10	rs11593867	10	48443093	G	A	−0.24	0.04	9.47×10^{-9}
GDF15	rs199580670	19	18475747	TGGC GCGG GGGG CTCA AAAC GGG	T	0.47	0.04	1.55×10^{-28}
GFRA1	rs11197613	10	118026217	G	T	−0.38	0.06	2.37×10^{-9}
GFRAL	rs6929594	6	55218980	G	A	0.61	0.04	1.91×10^{-50}
GGH	rs34223853	8	63953538	CA	C	0.46	0.04	4.12×10^{-28}
GKN2	rs13008230	2	69154583	T	G	−0.56	0.10	4.82×10^{-8}
GLCE	rs3865014	15	69561518	G	A	1.13	0.04	6.42×10^{-133}
GLIPR1	rs2242435	12	75874913	G	A	−0.22	0.04	3.99×10^{-8}
GLO1	rs34637217	6	38657463	C	T	−0.23	0.04	1.25×10^{-8}
GLRX2	rs74540121	1	193177917	T	A	0.25	0.04	2.51×10^{-12}
GNLY	rs370615710	2	85932002	GT	G	0.88	0.05	6.04×10^{-52}
GNRH1	rs13261573	8	25248615	G	A	−0.31	0.05	1.49×10^{-8}
GNRH2	rs8184100	20	3026415	C	T	−1.22	0.05	3.95×10^{-99}
GOLM1	rs148272757	9	88714593	C	A	0.45	0.05	5.14×10^{-17}
GP6	rs1613662	19	55536595	G	A	0.44	0.05	4.44×10^{-19}
GPC1	rs60515008	2	241408434	T	C	0.75	0.06	2.20×10^{-30}

GPC5	rs9523326	13	92052108	G	A	−0.30	0.03	2.47×10^{-24}
GPC6	rs1535692	13	95034749	G	A	−0.45	0.05	1.26×10^{-17}
GPNMB	rs858275	7	23294144	T	C	−0.80	0.03	5.35×10^{-95}
GRN	rs5848	17	42430244	C	T	−0.33	0.04	7.79×10^{-15}
GRP	rs8091691	18	56827121	G	A	−0.32	0.05	6.10×10^{-12}
GSN	rs76098787	9	124047836	T	C	0.92	0.07	3.36×10^{-33}
GSTM1	rs599363	1	110218486	G	T	−0.86	0.07	2.42×10^{-32}
GSTM3	rs12137743	1	110253555	C	T	−0.65	0.04	4.67×10^{-48}
GSTO1	rs1147611	10	106025258	G	T	−1.06	0.03	5.37×10^{-163}
GSTP1	rs1695	11	67352689	A	G	−0.79	0.03	1.64×10^{-106}
GSTT2B	rs5751777	22	24267047	C	T	−0.32	0.04	5.28×10^{-13}
GXYLT1	rs11181303	12	42437801	A	T	−0.34	0.06	8.50×10^{-9}
GZMB	rs8192918	14	25101878	C	CT	−0.97	0.04	5.96×10^{-85}
GZMM	rs3760874	19	544315	A	G	0.39	0.05	4.21×10^{-14}
H6PD	rs7555568	1	9292606	G	A	−0.60	0.04	1.35×10^{-39}
HAPLN1	rs13160212	5	83219474	T	C	1.07	0.03	8.74×10^{-145}
HAPLN4	rs2965187	19	19522970	T	A	0.24	0.03	1.12×10^{-18}
HAVCR2	rs6873507	5	156529747	A	G	−0.74	0.04	5.99×10^{-67}
HDGF	rs12145743	1	156700651	T	G	−0.29	0.03	3.18×10^{-18}
HGF	rs10248271	7	81417306	T	G	−0.25	0.04	1.64×10^{-8}
HGFAC	rs2498323	4	3451109	G	A	−1.28	0.06	7.43×10^{-82}
HIBCH	rs291466	2	191184475	A	G	0.62	0.03	7.34×10^{-72}
HK2	rs640944	2	75046839	T	G	−0.22	0.04	1.73×10^{-8}
HLA-DQA2	rs557011	6	32587013	C	T	0.53	0.04	1.72×10^{-42}
HMOX2	rs79848476	16	4829602	T	C	0.37	0.07	2.74×10^{-8}
HP	rs217184	16	72105965	T	C	0.82	0.05	8.95×10^{-46}
HRG	rs9898	3	186390627	C	T	0.47	0.04	6.26×10^{-27}
HSD17B14	rs35299026	19	49318380	G	A	−0.97	0.10	4.23×10^{-22}
HSP90B1	rs1177457	12	104336127	C	T	1.02	0.02	6.31×10^{-199}
HYAL1	rs78138837	3	50344178	A	G	0.72	0.07	7.47×10^{-22}
IBSP	rs2627692	4	88649369	C	T	−0.27	0.05	4.58×10^{-8}
ICAM1	rs5498	19	10395683	A	G	−0.70	0.03	1.32×10^{-106}
ICAM4	rs901886	19	10402131	T	C	0.16	0.03	4.22×10^{-9}

ICOSLG	rs112451903	21	45683369	A	ACTG	−0.30	0.04	4.51×10^{-14}
IDUA	rs56079856	4	994011	G	T	0.90	0.06	1.62×10^{-49}
IFNAR1	rs2257167	21	34715699	G	C	−1.16	0.03	3.14×10^{-174}
IFNL1	rs30461	19	39789115	A	G	0.43	0.06	3.12×10^{-11}
IFNL3	rs28416813	19	39735644	C	G	−0.32	0.04	8.34×10^{-15}
IFNLR1	rs10903035	1	24481940	G	A	0.25	0.04	1.75×10^{-9}
IGDCC3	rs525514	15	65615556	T	C	0.39	0.03	9.08×10^{-34}
IGDCC4	rs8034057	15	65789430	G	A	−0.55	0.09	3.70×10^{-10}
IGF2R	rs629849	6	160494409	A	G	0.99	0.05	2.63×10^{-75}
IGFBPL1	rs148002733	9	38852172	G	A	0.58	0.05	1.75×10^{-30}
IGFLR1	rs12459634	19	36230174	T	C	−0.64	0.04	5.28×10^{-47}
IGHG1	rs57446317	14	106206565	C	G	0.81	0.07	1.93×10^{-29}
IGHG2	rs57446317	14	106206565	C	G	0.81	0.07	1.93×10^{-29}
IGHG3	rs57446317	14	106206565	C	G	0.81	0.07	1.93×10^{-29}
IGHG4	rs57446317	14	106206565	C	G	0.81	0.07	1.93×10^{-29}
IGSF11	rs2903250	3	118649060	G	T	0.81	0.03	9.25×10^{-138}
IL10RA	rs3135932	11	117864063	A	G	−0.57	0.05	3.21×10^{-24}
IL10RB	rs2515717	21	34662282	G	A	0.52	0.04	5.01×10^{-31}
IL12B	rs2546890	5	158759900	A	G	0.51	0.05	2.20×10^{-27}
IL15RA	rs8177641	10	6016892	A	G	0.59	0.04	6.70×10^{-39}
IL16	rs4778639	15	81600451	T	G	−0.69	0.09	2.88×10^{-15}
IL17D	rs2314714	13	21270132	C	A	−0.53	0.09	2.32×10^{-9}
IL17RA	rs3827279	22	17595929	G	C	0.72	0.05	6.31×10^{-44}
IL17RB	rs62252978	3	53949262	T	C	0.37	0.06	2.99×10^{-9}
IL17RC	rs6765503	3	9964826	A	G	0.39	0.04	1.55×10^{-25}
IL17RD	rs35934471	3	57145830	C	CA	−1.02	0.03	2.61×10^{-140}
IL18R1	rs12996505	2	102931802	A	G	−1.01	0.02	1.64×10^{-194}
IL1R1	rs11689480	2	102674501	G	A	0.32	0.04	1.40×10^{-18}
IL1RAP	rs6444442	3	190346060	A	G	−1.45	0.05	1.61×10^{-128}
IL1RL1	rs7568913	2	102920037	T	C	−1.05	0.03	2.86×10^{-148}
IL1RL2	rs1922292	2	102829013	C	A	−0.39	0.04	3.23×10^{-26}
IL1RN	rs56341434	2	113868990	T	C	−0.25	0.04	4.45×10^{-9}
IL20	rs1150258	1	207074905	T	C	−0.21	0.03	4.97×10^{-10}

IL22	rs4144961	12	68656647	T	A	0.69	0.04	3.51×10^{-69}
IL27RA	rs35026308	19	14153293	T	C	−1.05	0.04	4.14×10^{-118}
IL2RB	rs228953	22	37531436	G	A	0.30	0.04	1.43×10^{-12}
IL34	rs35794088	16	70666796	C	T	−0.52	0.04	1.35×10^{-30}
IL5RA	rs7619345	3	3138841	G	A	0.51	0.05	6.27×10^{-27}
IL6	rs10274260	7	22844846	A	G	0.44	0.05	1.22×10^{-17}
IL6R	rs2228145	1	154426970	A	C	1.06	0.03	1.89×10^{-194}
IL6ST	rs35257381	5	55318612	A	T	0.20	0.03	1.52×10^{-10}
IL9	rs31551	5	135275330	G	A	0.56	0.04	1.08×10^{-43}
IMPAD1	rs62511997	8	57859634	C	T	−0.42	0.04	1.85×10^{-21}
INPP5B	rs579689	1	38256166	G	A	−0.52	0.04	1.91×10^{-36}
INSR	rs4804774	19	7772173	G	C	0.19	0.03	1.91×10^{-9}
IRF2	rs793785	4	185368274	A	C	0.33	0.05	5.73×10^{-12}
ISG15	rs1921	1	949608	G	A	0.41	0.03	5.92×10^{-36}
ITGB5	rs34410053	3	124442738	G	T	0.30	0.05	1.80×10^{-9}
ITIH4	rs34092621	3	52887861	A	AT	0.22	0.04	8.61×10^{-9}
ITIH5	rs41298373	10	7622009	G	A	−0.96	0.06	1.02×10^{-49}
JAM2	rs11909849	21	27168099	C	G	−0.19	0.03	9.08×10^{-11}
JAM3	rs6590735	11	134022087	G	T	0.19	0.03	2.03×10^{-8}
JAML	rs1540191	11	118090432	G	A	1.19	0.02	2.41×10^{-240}
KDR	rs34495369	4	56122001	G	A	−0.31	0.04	3.99×10^{-13}
KIRREL3	rs11220650	11	126760652	A	G	−0.20	0.02	5.67×10^{-26}
KLK10	rs62115757	19	51521821	T	G	−1.02	0.04	7.27×10^{-103}
KLK11	rs1048328	19	51527364	G	A	−1.39	0.06	8.81×10^{-87}
KLK12	rs3745540	19	51535130	A	G	−0.24	0.04	2.48×10^{-8}
KLK13	rs2569476	19	51569548	C	T	−0.51	0.08	3.03×10^{-11}
KLK14	rs3810091	19	51568690	C	T	0.37	0.06	4.55×10^{-10}
KLK15	rs5519	19	51322312	T	C	−0.65	0.04	4.41×10^{-45}
KLK7	rs1654526	19	51482459	G	A	0.93	0.05	1.25×10^{-56}
KLK8	rs2659074	19	51495579	T	G	−0.34	0.04	2.90×10^{-14}
KLKB1	rs2304595	4	187172280	G	A	0.35	0.04	2.78×10^{-19}
KLRB1	rs2241006	12	9748209	T	A	−0.71	0.04	3.06×10^{-60}
KNG1	rs5030049	3	186450863	T	C	−0.91	0.06	7.80×10^{-51}

KYNU	rs12477146	2	143793814	G	A	−0.32	0.05	9.25×10^{-12}
LAMC2	rs2276543	1	183155305	G	A	1.24	0.03	7.80×10^{-208}
LAYN	rs663763	11	111422217	C	T	0.61	0.03	6.42×10^{-89}
LBP	rs2232613	20	36997655	C	T	−1.04	0.07	2.21×10^{-40}
LCT	rs4988235	2	136608646	G	A	0.98	0.04	1.88×10^{-114}
LCTL	rs7403574	15	66857290	C	T	−0.40	0.06	1.45×10^{-11}
LEAP2	rs59414721	5	132322505	T	TG	0.41	0.05	3.64×10^{-16}
LECT2	rs31530	5	135282630	C	T	−0.33	0.03	3.86×10^{-22}
LEPR	rs12077336	1	66069986	G	T	−1.38	0.03	4.25×10^{-188}
LGALS2	rs6000806	22	37971179	T	C	−0.25	0.04	2.09×10^{-10}
LGALS3	rs76426991	14	55600939	G	A	−1.22	0.07	2.43×10^{-64}
LGALS8	rs34299988	1	236701748	C	T	0.67	0.04	6.48×10^{-66}
LGALS9	rs4239242	17	25974258	T	C	−0.33	0.04	8.74×10^{-16}
LGMN	rs9791	14	93170993	C	T	0.27	0.04	8.19×10^{-11}
LHB	rs78537284	19	49515363	G	A	−0.96	0.07	2.60×10^{-36}
LIFR	rs327287	5	38642552	G	T	0.18	0.03	2.41×10^{-10}
LILRA4	rs79828899	19	54853749	C	T	1.18	0.08	8.28×10^{-42}
LILRA6	rs11668526	19	54749060	C	T	0.64	0.04	3.27×10^{-42}
LILRB1	rs10427127	19	55143982	T	C	−1.55	0.07	1.12×10^{-86}
LILRB2	rs7247451	19	54782704	G	C	0.62	0.04	3.33×10^{-50}
LILRB5	rs12975366	19	54759361	T	C	−1.07	0.03	5.53×10^{-180}
LMAN2L	rs72809820	2	97360079	C	T	0.29	0.02	1.05×10^{-29}
LMOD1	rs2644112	1	201806106	T	C	−0.23	0.04	1.83×10^{-8}
LPO	rs7219860	17	56321271	G	A	−0.48	0.05	8.30×10^{-23}
LRIT2	rs4562751	10	85978723	T	A	0.41	0.05	1.47×10^{-13}
LRP11	rs7763718	6	150186534	T	G	0.39	0.02	1.11×10^{-77}
LRP12	rs28627996	8	105585028	C	T	−0.22	0.04	7.06×10^{-9}
LRP2	rs10201691	2	170069028	G	A	0.43	0.07	1.27×10^{-10}
LRP8	rs79395289	1	53734037	TG	T	0.36	0.02	2.43×10^{-68}
LRRC15	rs6799819	3	194089549	T	C	0.70	0.07	1.37×10^{-23}
LRRC32	rs1320644	11	76370187	G	A	−0.25	0.04	4.58×10^{-9}
LRRN1	rs35362954	3	3887508	C	G	−0.54	0.05	5.60×10^{-24}
LTF	rs55950019	3	46511644	T	A	1.11	0.03	3.78×10^{-173}

LY86	rs7757934	6	6578927	G	A	0.47	0.05	2.46×10^{-20}
LY9	rs1333064	1	160763600	G	A	0.33	0.05	1.32×10^{-12}
LY96	rs2929505	8	74879827	C	T	−0.29	0.05	1.17×10^{-8}
LYPLAL1	rs147082275	1	219454644	TAAA G	T	0.41	0.07	7.92×10^{-10}
LYZ	rs4761234	12	69732105	T	C	−0.74	0.03	4.58×10^{-90}
MAGI2	rs7779312	7	78116661	G	A	0.10	0.02	2.98×10^{-10}
MAN2B2	rs2301790	4	6600012	A	G	1.14	0.03	2.68×10^{-167}
MANBA	rs223492	4	103675108	G	C	0.38	0.04	6.00×10^{-24}
MANEA	rs35772543	6	96053922	T	A	−1.59	0.05	3.36×10^{-142}
MANF	rs6778196	3	51770429	C	T	−0.38	0.06	3.96×10^{-10}
MANSC4	rs9668702	12	27919491	T	G	0.89	0.04	4.31×10^{-87}
MAPK13	rs12210904	6	36098191	C	A	0.35	0.04	2.78×10^{-15}
MATN4	rs11086956	20	43924106	T	C	−0.59	0.04	1.85×10^{-43}
MAX	rs10143198	14	65555471	C	T	−0.23	0.03	4.07×10^{-11}
MBL2	rs7096206	10	54531685	G	C	0.79	0.05	5.04×10^{-57}
MCEE	rs4852762	2	71373036	C	T	−0.43	0.04	1.92×10^{-24}
MESD	rs11855057	15	81282144	C	G	−0.39	0.05	2.84×10^{-15}
METTL24	rs13218597	6	110679736	A	G	0.44	0.03	9.92×10^{-36}
MFAP2	rs2284746	1	17306675	C	G	0.64	0.04	4.24×10^{-48}
MGP	rs2430737	12	15035563	T	C	0.35	0.04	1.40×10^{-16}
MIA	rs2607412	19	41268243	A	G	0.73	0.08	5.47×10^{-19}
MICA	rs9281428	6	31378227	C	CT	−1.08	0.03	8.03×10^{-154}
MICB	rs9266244	6	31325692	G	A	0.86	0.05	8.03×10^{-49}
MIF	rs2070766	22	24237221	C	G	−0.34	0.05	3.70×10^{-12}
MLN	rs55775340	6	33751784	C	G	−0.34	0.05	2.53×10^{-10}
MMEL1	rs4648652	1	2535758	A	G	1.16	0.03	5.28×10^{-167}
MMP10	rs486055	11	102650424	C	T	−0.67	0.06	7.72×10^{-28}
MMP12	rs114176245	11	102719534	C	T	−0.54	0.06	2.79×10^{-16}
MMP2	rs1561220	16	55504568	A	G	0.56	0.06	3.20×10^{-21}
MMP7	rs79643393	11	102410668	C	T	1.25	0.08	9.51×10^{-47}
MMP8	rs2155052	11	102595666	C	G	−1.32	0.06	8.31×10^{-84}
MPIG6B	rs11575845	6	31692386	C	G	−1.14	0.08	7.17×10^{-40}

MRC1	rs201259350	10	17852064	TAGA<br>A	T	−0.38	0.05	5.62×10^{-14}
MRC2	rs138105166	17	60704973	TCCT<br>CCCT<br>CCGC	T	−0.33	0.04	9.58×10^{-15}
MSMB	rs10993994	10	51549496	T	C	0.85	0.03	1.37×10^{-117}
MSMP	rs10758322	9	35776190	C	T	−0.59	0.04	9.41×10^{-52}
MST1	rs11130213	3	49712297	C	T	−1.12	0.03	4.99×10^{-187}
MTHFS	rs149183525	15	80168321	AAAA<br>T	A	−0.66	0.04	3.07×10^{-48}
MXRA7	rs720782	17	74682602	C	T	−0.35	0.03	1.05×10^{-36}
MXRA8	rs307346	1	1260733	A	C	0.39	0.07	2.22×10^{-8}
NAAA	rs7686066	4	76838858	A	T	−0.87	0.04	2.73×10^{-88}
NAGK	rs2287327	2	71297982	C	T	−0.29	0.03	1.41×10^{-17}
NAGPA	rs12599777	16	5079466	A	G	−0.36	0.04	2.32×10^{-20}
NBL1	rs2854108	1	19973920	A	G	−0.32	0.02	1.32×10^{-45}
NCAM2	rs2009029	21	22855892	A	G	0.32	0.03	4.61×10^{-21}
NCR1	rs77273876	19	55400971	G	A	1.23	0.08	5.52×10^{-44}
NCR3	rs986475	6	31556709	A	G	−0.56	0.06	6.56×10^{-22}
NDNF	rs6840113	4	121936507	A	G	0.49	0.04	5.48×10^{-31}
NDST1	rs2545341	5	149914401	T	C	0.22	0.03	1.01×10^{-14}
NELL1	rs79474191	11	20953407	C	T	1.16	0.08	2.57×10^{-45}
NID1	rs9662380	1	236178606	G	A	−0.34	0.05	4.20×10^{-13}
NID2	rs140170580	14	52494307	T	TA	−0.70	0.05	3.36×10^{-40}
NMB	rs34452033	15	85221993	A	G	−0.33	0.04	5.02×10^{-17}
NPNT	rs34712979	4	106819053	G	A	−0.35	0.05	8.20×10^{-12}
NPPB	rs12402728	1	11922148	A	G	0.53	0.05	8.81×10^{-28}
NPW	rs3785284	16	2074036	A	G	−0.78	0.04	1.19×10^{-65}
NQO1	rs112668868	16	69713957	C	G	−1.00	0.04	2.79×10^{-93}
NQO2	rs2756078	6	3010103	G	A	0.98	0.04	9.61×10^{-102}
NRG1	rs10089448	8	32597355	C	A	−0.50	0.06	1.25×10^{-14}
NRG4	rs35468194	15	76349565	T	TA	1.26	0.03	2.96×10^{-169}
NRP1	rs2506149	10	33480713	C	T	−0.33	0.03	7.98×10^{-23}
NRP2	rs11678440	2	206548443	T	C	−0.17	0.03	4.32×10^{-8}
NT5C	rs111346516	17	73603700	G	A	−0.40	0.06	2.70×10^{-11}

NTM	rs12790269	11	131148713	G	A	0.13	0.02	6.33×10^{-15}
NTN1	rs940854	17	8934763	C	T	−0.28	0.04	2.32×10^{-13}
NTN4	rs7959545	12	96059957	G	C	0.80	0.05	5.31×10^{-46}
NTRK1	rs2365715	1	156615114	A	G	−0.43	0.04	1.01×10^{-22}
NUCB1	rs2017135	19	49383269	C	T	−0.44	0.06	3.37×10^{-12}
NUDCD3	rs306997	7	44538882	A	G	0.45	0.03	2.74×10^{-37}
NUDT9	rs58601962	4	88384477	A	AC	−0.51	0.06	3.06×10^{-18}
OAF	rs2508490	11	120099679	G	A	−0.87	0.05	1.72×10^{-58}
OAS1	rs10850097	12	113361117	C	T	−0.48	0.04	1.07×10^{-34}
OLFM1	rs546682	9	137970302	C	T	−0.22	0.03	4.97×10^{-12}
OLFM2	rs1862474	19	10096650	T	C	0.42	0.04	6.33×10^{-21}
OLFML3	rs3841011	1	114521683	A	AAAC	0.34	0.05	2.24×10^{-12}
OLR1	rs17808009	12	10311929	C	T	0.18	0.03	7.39×10^{-11}
OMD	rs117800660	9	95174368	A	T	−0.47	0.08	9.14×10^{-9}
OSCAR	rs254252	19	54594864	G	A	0.52	0.08	9.23×10^{-11}
OSMR	rs72732754	5	38999995	A	T	−0.31	0.04	1.19×10^{-11}
PAM	rs34274728	5	102306489	CT	C	−0.47	0.03	1.56×10^{-40}
PCBD1	rs2630336	10	72648422	G	T	−0.26	0.04	5.40×10^{-13}
PCDH10	rs1112105	4	133894412	T	G	0.21	0.03	2.30×10^{-14}
PCOLCE2	rs2707975	3	142611657	A	G	0.78	0.05	5.27×10^{-50}
PCSK1	rs6234	5	95728974	G	C	−0.83	0.03	9.39×10^{-122}
PCSK7	rs236910	11	117081974	A	G	−1.13	0.03	2.24×10^{-151}
PCSK9	rs41294819	1	55504586	A	G	−0.85	0.05	1.39×10^{-49}
PDCD1LG2	rs62556118	9	5515990	G	C	0.53	0.04	2.60×10^{-41}
PDCD5	rs4499344	19	33073431	G	A	0.25	0.03	1.17×10^{-13}
PDGFD	rs10444324	11	104095391	A	C	0.42	0.04	8.55×10^{-21}
PDGFRB	rs3816018	5	149508475	C	T	1.14	0.02	4.59×10^{-308}
PDGFRL	rs2720579	8	17432466	G	A	−0.83	0.05	1.06×10^{-51}
PDLIM4	rs4877	5	131607588	G	T	−0.38	0.05	2.34×10^{-14}
PENK	rs1877571	8	57426778	A	C	0.46	0.04	3.19×10^{-26}
PGD	rs72867415	1	10416602	G	A	−0.68	0.08	1.52×10^{-17}
PIGR	rs533494	1	207117575	C	T	0.42	0.05	1.57×10^{-16}
PILRA	rs1859788	7	99971834	A	G	−1.29	0.03	1.02×10^{-204}

PKDCC	rs10495892	2	42156206	G	C	0.24	0.04	4.13×10^{-8}
PLA2G2A	rs11573156	1	20306146	G	C	0.29	0.05	7.72×10^{-9}
PLA2G7	rs1421378	6	46703513	A	G	0.79	0.03	1.19×10^{-94}
PLA2R1	rs3749117	2	160885442	T	C	−0.97	0.03	3.91×10^{-122}
PLAUR	rs4760	19	44153100	A	G	−0.26	0.03	2.00×10^{-14}
PLD5	rs72761220	1	242267176	A	T	0.32	0.05	2.30×10^{-12}
PLEK	rs3816281	2	68607947	G	T	0.36	0.04	4.56×10^{-17}
PLOD2	rs1707466	3	145850024	G	A	−0.36	0.04	3.70×10^{-20}
PLXDC2	rs7094178	10	20105223	G	A	0.27	0.03	3.81×10^{-18}
PLXNB2	rs28573806	22	50727792	T	C	−1.22	0.02	2.23×10^{-308}
PLXNC1	rs12313790	12	94632933	C	A	−0.39	0.06	5.99×10^{-10}
PNLIPRP2	rs7910135	10	118398046	C	A	0.82	0.03	4.86×10^{-93}
POFUT1	rs76143353	20	30815755	C	T	−1.11	0.09	7.73×10^{-33}
POGLUT1	rs6794833	3	119191398	T	C	−0.65	0.04	2.74×10^{-62}
POGLUT3	rs111559044	11	108254454	G	C	−0.69	0.09	2.89×10^{-13}
POMC	rs3754861	2	25393722	A	C	0.37	0.06	7.04×10^{-11}
POMGNT2	rs2002182	3	43147538	T	G	−0.26	0.04	8.36×10^{-12}
POSTN	rs9547910	13	38069999	G	A	−0.58	0.04	3.79×10^{-43}
PPID	rs6856561	4	159621185	A	T	0.43	0.03	1.29×10^{-31}
PPIE	rs1046988	1	40219065	C	T	−0.45	0.03	4.74×10^{-42}
PPIF	rs11002931	10	81094251	A	C	−0.47	0.04	3.83×10^{-24}
PPT1	rs112524162	1	40576715	G	T	−0.93	0.10	1.26×10^{-20}
PRKCSH	rs160839	19	11557697	T	C	−0.25	0.04	2.07×10^{-11}
PROS1	rs5013930	3	93679707	T	C	0.35	0.05	1.28×10^{-10}
PRTN3	rs6510982	19	845535	C	G	0.33	0.04	2.47×10^{-14}
PSG3	rs4030933	19	43237764	T	G	−0.42	0.04	1.16×10^{-24}
PSG9	rs140022160	19	43784407	C	CCCTT	0.40	0.05	2.52×10^{-13}
PSMB1	rs3734763	6	170885912	T	C	−0.36	0.03	6.32×10^{-37}
PSPN	rs6510895	19	6391847	C	T	0.43	0.04	1.23×10^{-20}
PTGFRN	rs4233450	1	117490261	G	T	0.87	0.04	1.08×10^{-78}
PTGR1	rs56912703	9	114357659	T	A	−0.42	0.04	8.62×10^{-21}
PTH	rs78157059	11	13691962	C	G	−0.45	0.07	9.70×10^{-12}
PTHLH	rs10843110	12	28289141	T	G	−0.29	0.05	3.76×10^{-10}

PTPN4	rs11896956	2	120135332	A	G	−0.49	0.06	4.95×10^{-17}
PTPRU	rs212297	1	29723516	C	T	−0.42	0.03	8.55×10^{-37}
PXYLP1	rs6786821	3	140999276	C	G	0.47	0.04	1.14×10^{-25}
QDPR	rs4698602	4	17519712	G	A	−0.36	0.04	2.29×10^{-15}
QPCT	rs12467820	2	37572806	G	T	−0.46	0.03	2.63×10^{-51}
QPCTL	rs2110574	19	46197421	C	T	0.16	0.03	1.27×10^{-9}
QSOX1	rs12371	1	180163390	A	G	−0.52	0.05	3.82×10^{-28}
QSOX2	rs3758199	9	139097645	C	T	−0.25	0.03	2.63×10^{-14}
RABEPK	rs147332108	9	127911299	TA	T	1.05	0.04	1.72×10^{-107}
RAET1L	rs6557222	6	150300478	A	G	−0.46	0.05	3.67×10^{-18}
RARRES1	rs6441224	3	158450417	T	C	0.42	0.05	8.46×10^{-19}
RARRES2	rs28432021	7	150046235	G	C	0.72	0.05	1.41×10^{-45}
RBP7	rs3811458	1	10057203	G	T	−0.61	0.06	1.90×10^{-22}
REG1A	rs76841471	2	79330169	T	G	0.50	0.08	2.91×10^{-10}
REG3G	rs436758	2	79247510	T	C	0.67	0.04	2.84×10^{-45}
REG4	rs58163904	1	120365410	GCA	G	−0.45	0.05	5.07×10^{-19}
RGMA	rs11858429	15	93844067	C	T	0.21	0.03	2.87×10^{-11}
RIDA	rs7846130	8	99109401	A	G	−0.23	0.02	1.61×10^{-24}
RMDN1	rs10107571	8	87518985	G	T	−1.04	0.02	6.71×10^{-229}
RNASE2	rs2233859	14	21359808	C	A	0.63	0.04	4.16×10^{-59}
RNASE3	rs11299601	14	21349400	AG	A	−0.67	0.05	3.39×10^{-38}
RNASE4	rs944438	14	21164627	A	G	−0.32	0.04	4.98×10^{-15}
RNASE6	rs1045922	14	21250124	G	A	1.23	0.03	8.93×10^{-240}
ROBO2	rs74506149	3	77580700	A	C	−0.28	0.04	3.28×10^{-12}
ROBO3	rs55706177	11	124746180	C	T	0.38	0.05	1.44×10^{-14}
ROR1	rs61765448	1	64611828	C	T	−0.45	0.04	2.18×10^{-24}
ROR2	rs4744097	9	94543365	G	A	0.25	0.03	7.41×10^{-15}
RPE	rs2887872	2	210878117	C	T	−0.25	0.03	6.63×10^{-19}
RPN1	rs2712419	3	128340896	T	C	−0.51	0.02	1.79×10^{-79}
RRM2B	rs2305832	8	103266805	C	A	0.52	0.06	2.43×10^{-15}
RSPO2	rs593872	8	108941705	G	A	0.54	0.04	2.56×10^{-42}
RSPO4	rs4813028	20	1096630	T	C	0.36	0.06	2.49×10^{-8}
S100A4	rs1005436	1	153521932	G	A	0.55	0.07	3.96×10^{-15}

S100A7	rs3006490	1	153369009	A	C	−1.07	0.09	1.86×10^{-28}
SAA1	rs10690148	11	18293504	C	CAACTT	−0.94	0.04	4.53×10^{-87}
SAT2	rs11078700	17	7511936	A	G	−0.55	0.07	1.50×10^{-14}
SCARA3	rs2640734	8	27532654	C	T	−0.31	0.05	3.08×10^{-9}
SCARB2	rs78466149	4	76393394	G	A	−0.31	0.06	4.95×10^{-8}
SCARF1	rs145220082	17	1545567	T	C	1.44	0.05	9.67×10^{-116}
SCARF2	rs10606665	22	20776973	AACC	A	0.32	0.05	1.55×10^{-9}
SCG3	rs1456297	15	51964210	C	A	0.34	0.04	4.77×10^{-14}
SCGN	rs4419666	6	25693274	C	T	0.40	0.05	5.65×10^{-18}
SCT	rs79177795	11	644748	C	G	0.70	0.06	8.66×10^{-26}
SCUBE1	rs138993	22	43610207	A	G	0.25	0.04	1.95×10^{-8}
SELL	rs2223286	1	169665632	T	C	−0.66	0.04	3.99×10^{-51}
SELP	rs6128	1	169562904	C	T	−0.66	0.05	3.16×10^{-33}
SEMA3E	rs3801487	7	83033915	T	C	−1.19	0.05	1.99×10^{-109}
SEMA3G	rs34135146	3	52313432	G	C	−0.29	0.04	1.73×10^{-11}
SEMA4D	rs45464494	9	91994433	C	T	−0.79	0.05	2.89×10^{-47}
SERPINA1	rs1243167	14	94841641	G	A	0.28	0.05	2.40×10^{-8}
SERPINA4	rs10140765	14	95023389	C	T	0.36	0.04	1.57×10^{-16}
SERPINA5	rs885786	14	95050496	C	G	0.40	0.04	2.43×10^{-20}
SERPINA9	rs12896629	14	94940361	G	T	0.16	0.03	3.39×10^{-9}
SERPINB1	rs386713	6	2842139	C	T	−0.40	0.04	2.39×10^{-27}
SERPIND1	rs6004023	22	21065285	A	G	0.38	0.04	1.80×10^{-19}
SERPINE1	rs2227674	7	100776208	A	G	0.32	0.05	3.88×10^{-9}
SERPINF1	rs12450371	17	1667674	C	T	−0.51	0.04	1.93×10^{-29}
SERPING1	rs11229075	11	57391023	A	C	0.84	0.04	1.20×10^{-82}
SFRP4	rs17236800	7	37944375	A	G	−0.33	0.05	8.07×10^{-10}
SHBG	rs12452603	17	7504977	T	C	0.30	0.05	1.19×10^{-8}
SIGLEC12	rs4801871	19	52002317	G	C	−1.35	0.04	1.01×10^{-141}
SIGLEC14	rs147859411	19	52130148	T	TTTTTG	−1.12	0.06	2.60×10^{-62}
SIGLEC5	rs147859411	19	52130148	T	TTTTTG	−1.16	0.06	5.75×10^{-78}
SIGLEC6	rs2305771	19	52033572	T	C	0.31	0.05	6.18×10^{-11}
SIGLEC7	rs12983058	19	51642784	G	T	1.20	0.03	1.44×10^{-170}

SIGLEC9	rs2075803	19	51628529	A	G	−1.18	0.02	1.09×10^{-240}
SIRPA	rs6075340	20	1894335	G	A	−1.17	0.03	5.81×10^{-196}
SIRPB1	rs2318043	20	1596473	A	G	1.27	0.04	4.68×10^{-145}
SIRPG	rs17855609	20	1895813	A	T	−0.60	0.05	1.87×10^{-34}
SLAMF7	rs11581248	1	160720074	C	T	−1.21	0.06	2.92×10^{-74}
SMOC1	rs12101270	14	70463688	T	C	−0.41	0.04	2.46×10^{-19}
SMPDL3A	rs9385271	6	123125411	T	C	−0.82	0.04	3.29×10^{-72}
SOD3	rs11938550	4	24726263	G	C	−0.68	0.04	9.29×10^{-54}
SORBS3	rs1047030	8	22428708	A	G	−0.36	0.05	8.98×10^{-12}
SORCS2	rs3892041	4	7314962	A	C	0.36	0.05	1.02×10^{-12}
SOST	rs9899889	17	41823225	T	G	−0.55	0.04	2.32×10^{-39}
SPARCL1	rs17012853	4	88454184	C	T	−0.35	0.05	2.82×10^{-13}
SPAST	rs79204217	2	31902450	T	C	0.23	0.04	2.04×10^{-8}
SPATA20	rs8076632	17	48625928	C	G	−0.50	0.04	2.73×10^{-33}
SPINK2	rs781538	4	57685669	C	T	0.52	0.04	4.30×10^{-41}
SPINK6	rs765776722	5	147592991	T	TG	−1.39	0.07	2.38×10^{-75}
SPINT1	rs17658212	15	41145919	C	T	−0.68	0.08	1.24×10^{-16}
SPINT2	rs71354995	19	38791841	A	G	−1.20	0.03	1.99×10^{-207}
SPINT3	rs6017593	20	44147814	A	G	0.25	0.03	1.07×10^{-13}
SPOCK2	rs1245550	10	73844081	A	G	−0.56	0.04	3.06×10^{-39}
SPON1	rs1528661	11	13971798	T	C	−0.43	0.03	1.78×10^{-31}
SPON2	rs878323	4	1169813	G	T	−0.59	0.04	1.73×10^{-39}
ST3GAL1	rs2142306	8	134470631	T	C	0.29	0.04	1.72×10^{-15}
ST3GAL6	rs113325534	3	98786038	C	T	−1.45	0.08	9.24×10^{-64}
ST6GALNAC1	rs2286595	17	74621635	C	T	0.20	0.03	1.78×10^{-8}
ST6GALNAC2	rs10852772	17	74580421	G	A	−0.48	0.05	4.92×10^{-20}
ST8SIA2	rs34728194	15	93064556	A	C	−0.23	0.04	6.50×10^{-9}
STC1	rs138694283	8	23818092	C	CT	0.54	0.09	2.57×10^{-9}
STX8	rs11078801	17	9337245	G	C	0.12	0.02	1.00×10^{-11}
SVEP1	rs74308641	9	113313412	G	A	−0.58	0.08	1.08×10^{-13}
TAC1	rs10272589	7	96896639	G	A	0.65	0.06	3.60×10^{-29}
TAPBP	rs469064	6	33250476	C	A	−0.32	0.04	3.17×10^{-16}

TAPBPL	rs2041387	12	6562823	C	T	1.32	0.03	5.04×10^{-239}
TAX1BP3	rs2873624	17	3563963	C	G	−0.25	0.04	1.86×10^{-10}
TBCE	rs868815	1	235584543	C	G	−0.18	0.03	3.87×10^{-11}
TBL2	rs76029572	7	72992858	C	G	1.28	0.08	2.03×10^{-51}
TCN2	rs12169610	22	31022590	C	T	−0.72	0.06	6.36×10^{-28}
TDGF1	rs80045772	3	46619238	T	A	1.22	0.03	9.62×10^{-237}
TEK	rs16911092	9	27142444	A	G	0.98	0.07	2.82×10^{-36}
TFF1	rs3761376	21	43787038	G	A	−0.32	0.05	4.15×10^{-9}
TGFB1	rs57948035	19	41882044	CAAA A	C	0.23	0.03	3.81×10^{-14}
TGFB2	rs7533619	1	219346409	C	T	−0.21	0.04	5.04×10^{-9}
TGFB3	rs11621464	14	76379798	C	G	0.43	0.06	8.35×10^{-14}
TGM3	rs214830	20	2321105	G	C	0.42	0.05	5.78×10^{-18}
THBS2	rs7341189	6	169622734	G	A	0.26	0.05	1.68×10^{-8}
THBS3	rs4072037	1	155162067	C	T	−0.34	0.04	2.53×10^{-14}
THBS4	rs256438	5	79366249	T	G	0.53	0.04	4.95×10^{-33}
THSD1	rs191768	13	53152704	T	C	−0.46	0.08	2.72×10^{-8}
TIE1	rs1199039	1	43784956	A	G	−0.24	0.03	1.68×10^{-12}
TIGAR	rs143427116	12	4430340	AGCC GGCC GGCT	A	0.60	0.07	1.37×10^{-15}
TLR4	rs4986791	9	120475602	C	T	−1.05	0.06	5.43×10^{-65}
TMED2	rs786430	12	124087988	G	C	−0.34	0.03	4.04×10^{-33}
TMEM106B	rs4721060	7	12267552	G	A	−0.37	0.03	2.15×10^{-42}
TMEM132A	rs55920775	11	60703777	G	A	0.45	0.03	2.41×10^{-57}
TMEM132C	rs7313554	12	128791042	T	C	0.16	0.02	6.27×10^{-11}
TMEM132D	rs12372807	12	129561332	G	A	0.22	0.04	1.25×10^{-8}
TMEM190	rs4806666	19	55888095	C	T	−1.14	0.03	2.15×10^{-172}
TMEM25	rs200268771	11	118446875	G	GT	−0.51	0.09	2.54×10^{-9}
TMEM9	rs2365296	1	201114196	C	T	−0.77	0.02	3.62×10^{-217}
TMPRSS5	rs11315692	11	113616733	TG	T	−0.62	0.04	2.06×10^{-51}
TNC	rs1138545	9	117835899	C	T	1.38	0.06	2.85×10^{-100}
TNFAIP6	rs34026490	2	152205792	T	C	0.33	0.04	5.93×10^{-15}
TNFRSF10B	rs4871844	8	22879734	T	C	0.45	0.03	1.36×10^{-41}
TNFRSF11A	rs884205	18	60054857	A	C	−0.43	0.03	1.93×10^{-41}

TNFRSF14	rs2281852	1	2490942	C	A	0.36	0.02	1.24×10^{-56}
TNFRSF18	rs12066716	1	1123434	T	A	−0.43	0.04	4.00×10^{-21}
TNFRSF19	rs3751364	13	24234517	A	G	0.27	0.04	4.70×10^{-11}
TNFRSF1B	rs616645	1	12240824	T	G	−0.25	0.04	7.23×10^{-9}
TNFRSF4	rs190796582	1	1167796	C	T	0.60	0.07	1.00×10^{-16}
TNFRSF8	rs3830887	1	12066218	T	TA	0.50	0.04	2.72×10^{-34}
TNFSF13B	rs1224148	13	108956794	A	G	0.40	0.05	5.00×10^{-14}
TNFSF15	rs7848647	9	117569046	T	C	−0.53	0.04	4.25×10^{-41}
TNFSF8	rs4979474	9	117695984	G	A	−0.26	0.04	8.36×10^{-9}
TNXB	rs201459441	6	31962708	AC	A	−0.62	0.09	1.35×10^{-11}
TPSAB1	rs112332886	16	1288563	G	A	1.20	0.07	5.25×10^{-50}
TPSB2	rs112332886	16	1288563	G	A	1.37	0.07	2.28×10^{-73}
TREM1	rs9462692	6	41262236	A	C	−0.72	0.05	1.67×10^{-47}
TRH	rs2670897	3	129699722	C	T	0.64	0.05	4.18×10^{-33}
TRIL	rs3735562	7	28996557	C	T	−0.47	0.05	1.06×10^{-20}
TXNDC15	rs3733897	5	134223593	A	G	1.10	0.06	3.45×10^{-72}
TXNRD1	rs11111979	12	104680782	C	G	−0.13	0.02	4.15×10^{-9}
TYMP	rs140522	22	50971266	T	C	0.29	0.03	8.16×10^{-21}
UCMA	rs2399954	10	13276368	G	A	−0.85	0.05	3.08×10^{-53}
ULBP2	rs6557222	6	150300478	A	G	−0.35	0.05	7.33×10^{-11}
ULBP3	rs12661513	6	150373839	C	A	−1.01	0.05	1.26×10^{-75}
UNC5C	rs10516960	4	96159677	T	G	0.15	0.02	2.78×10^{-9}
UST	rs1384668	6	149223723	C	G	−0.38	0.03	6.60×10^{-26}
VASN	rs886858	16	4422122	A	G	0.40	0.05	2.92×10^{-16}
VIT	rs11124542	2	36994439	A	C	0.50	0.03	1.17×10^{-50}
VSIR	rs9415993	10	73514911	G	C	0.57	0.04	3.79×10^{-42}
VTN	rs704	17	26694861	G	A	1.11	0.02	2.76×10^{-243}
VWA2	rs11196687	10	116059728	G	A	−0.75	0.05	6.72×10^{-41}
WARS1	rs4905953	14	100810426	G	A	0.36	0.04	6.37×10^{-20}
WFDC1	rs12448765	16	84425214	A	G	−0.30	0.05	4.00×10^{-11}
WFIKKN1	rs8062289	16	681284	C	T	0.80	0.05	5.91×10^{-59}
WFIKKN2	rs7225019	17	48915879	G	A	−1.06	0.04	7.23×10^{-121}
XCL1	rs1323532	1	168496272	G	T	0.27	0.04	1.41×10^{-9}

ZG16B	rs4785908	16	2879279	T	C	0.56	0.05	6.04×10^{-32}

RSID stands for Reference SNP Identifier, CHR stands for Chromosome, POS stands for Position in base pairs, REF is the Reference Allele, ALT is the Alternative Allele.

Supplementary Table 2 Mendelian randomization using low linkage disequilibrium (LD) threshold

Protein	Nr of IVs	Method	β	Nominal *P*-value	Colocalization PP.H4.abf	Horizontal Pleiotropy Test (MRPRESSO *P*-value)
GPNMB[a,b]	11	IVW	0.14	1.39×10^{-20}	0.94	0.65
FCGR2B[b]	17	IVW	0.07	2.43×10^{-16}	0.96	0.49
FCGR2A[b]	18	IVW	0.07	1.93×10^{-14}	0.95	0.26
CTSB[b]	12	IVW	−0.11	2.19×10^{-12}	0.22	0.47
CD38[b]	1	Wald ratio	−0.53	2.62×10^{-11}	0.45	NA
HP[b]	19	IVW	0.06	2.38×10^{-8}	0.01	0.60
LTF[b]	23	IVW	0.05	2.82×10^{-8}	0.04	0.65
HLA-DQA2[b,c]	33	IVW	−0.14	1.20×10^{-7}	0.03	0.00
HAVCR2[b]	13	IVW	−0.10	4.77×10^{-7}	0.30	0.67
CLEC3B[b]	4	IVW	−0.16	1.94×10^{-6}	0.29	0.97
BST1[b,c]	10	IVW	0.12	2.63×10^{-6}	0.24	0.00
HAPLN1[b]	15	IVW	0.06	7.24×10^{-6}	0.05	0.79
MANEA[b]	17	IVW	0.05	7.26×10^{-6}	0.10	0.94
NQO2[b]	11	IVW	0.05	7.63×10^{-6}	0.05	0.98
ARSA[a,b]	5	IVW	0.13	1.70×10^{-5}	0.58	0.62
LGALS3[b]	8	IVW	0.07	1.86×10^{-5}	0.02	0.50
PAM[b]	12	IVW	0.09	2.64×10^{-5}	0.08	0.47
IL1RL1[b]	26	IVW	0.03	2.75×10^{-5}	0.01	0.90
TPSAB1[b]	8	IVW	−0.06	3.22×10^{-5}	0.15	0.80
HSP90B1[b]	22	IVW	0.04	3.50×10^{-5}	0.03	0.19
GLCE[b]	14	IVW	0.05	4.84×10^{-5}	0.10	0.96
MANSC4[b]	18	IVW	0.05	5.20×10^{-5}	0.02	0.85
ICAM1[b]	8	IVW	−0.07	7.29×10^{-5}	0.32	0.74
RABEPK[b]	10	IVW	−0.05	7.33×10^{-5}	0.10	0.86
SIRPB1[b]	25	IVW	−0.03	9.11×10^{-5}	0.01	0.60

CLN5[a,b]	2	IVW	−0.15	1.19×10^{-4}	0.38	NA
IL9[b]	12	IVW	0.06	1.21×10^{-4}	0.05	0.96
PCSK7[b]	18	IVW	−0.04	1.21×10^{-4}	0.02	0.98
AGT[b]	4	IVW	0.12	1.77×10^{-4}	0.06	0.97
CD274[b]	16	IVW	0.08	1.96×10^{-4}	0.02	0.46
RBP7[b]	3	IVW	0.20	2.23×10^{-4}	0.09	NA
C4B[b,c,d]	101	IVW	0.02	2.85×10^{-4}	0.00	0.00
PLA2G7[b]	14	IVW	0.04	2.96×10^{-4}	0.05	0.57
C4A[b,c,d]	101	IVW	0.02	3.76×10^{-4}	0.00	0.00
ASIP[b]	3	IVW	−0.13	5.13×10^{-4}	0.17	NA
EGF[b]	3	IVW	−0.13	5.23×10^{-4}	0.11	NA
TPSB2[b]	10	IVW	−0.05	5.96×10^{-4}	0.15	0.27
RNASE3[b]	6	IVW	0.09	6.14×10^{-4}	0.01	0.26
ACP1[b]	15	IVW	0.04	6.25×10^{-4}	0.04	0.88
A4GALT[b]	2	IVW	−0.21	6.62×10^{-4}	0.11	NA
DSCAM[b]	3	IVW	−0.30	8.78×10^{-4}	0.19	NA
LCT[b,c]	25	IVW	0.04	8.91×10^{-4}	0.00	0.00
COLEC11[b]	8	IVW	−0.05	9.58×10^{-4}	0.03	0.61
SPOCK2[b]	8	IVW	0.08	0.0010	0.03	0.98
VWA2[b]	5	IVW	−0.11	0.0011	0.04	0.76
ADAMTS4[b]	8	IVW	−0.06	0.0013	0.19	0.36
RNASE2[b]	10	IVW	0.06	0.0013	0.02	0.61
PRTN3[b]	2	IVW	0.17	0.0013	0.06	NA
RPN1[b]	10	IVW	0.08	0.0013	0.02	0.57
PDCD1LG2[b]	12	IVW	−0.09	0.0013	0.04	0.07
ADGRE2[b]	10	IVW	−0.05	0.0013	0.01	0.87
MPIG6B[b]	18	IVW	−0.05	0.0014	0.00	0.18

TAPBPL[b]	10	IVW	−0.03	0.0014	0.03	0.50
SIGLEC9[b]	19	IVW	0.03	0.0015	0.02	0.99
LRP12[a,b]	1	Wald ratio	0.73	0.0017	0.66	NA
DNAJC30[b]	3	IVW	−0.09	0.0018	0.03	NA
CCL15[b]	3	IVW	−0.08	0.0019	0.12	NA
VTN[b]	22	IVW	−0.03	0.0022	0.01	0.75
NUCB1[b]	3	IVW	−0.12	0.0023	0.05	NA
TRH[b]	4	IVW	−0.08	0.0023	0.07	0.75
POSTN[b]	8	IVW	−0.07	0.0028	0.02	0.76
PLXNB2[a,b]	36	IVW	−0.02	0.0029	0.08	0.09
IL18R1[b]	23	IVW	0.03	0.0029	0.01	0.79
IDUA[b]	3	IVW	0.10	0.0033	0.00	NA
CFD[b]	10	IVW	−0.05	0.0035	0.01	0.58
GGH[a,b]	4	IVW	0.08	0.0035	0.02	0.49
FGFRL1[b]	5	IVW	0.13	0.0041	0.00	0.07
LMAN2L[b]	3	IVW	−0.15	0.0045	0.03	NA
TXNDC15	6	IVW	−0.05	0.0048	0.12	0.46
KNG1	6	IVW	0.05	0.0050	0.03	0.80
EPHA1	6	IVW	0.05	0.0050	0.08	0.98
CCL23	2	IVW	0.10	0.0054	0.03	NA
JAML	13	IVW	−0.02	0.0055	0.03	0.86
CLEC7A	5	IVW	0.05	0.0059	0.09	0.87
HRG	2	IVW	−0.08	0.0060	0.09	NA
NUDT9	4	IVW	−0.08	0.0070	0.03	0.87
CHI3L2	11	IVW	−0.04	0.0072	0.04	0.45
OAS1	5	IVW	0.08	0.0080	0.02	0.41
TNFRSF11A	12	IVW	−0.06	0.0082	0.02	0.99

NDNF	4	IVW	−0.10	0.0083	0.01	0.32
INPP5B	6	IVW	−0.08	0.0096	0.01	0.43
GRN[a]	1	Wald ratio	−0.19	0.0097	0.22	NA
WFIKKN2	10	IVW	0.03	0.010	0.00	0.70
ROR2	4	IVW	0.13	0.010	0.01	0.42
H6PD	6	IVW	−0.07	0.010	0.05	0.92
GFRAL	8	IVW	0.05	0.012	0.04	0.97
BTN2A1	12	IVW	0.07	0.013	0.00	0.19
MMP2	5	IVW	−0.08	0.014	0.03	0.96
PGD	2	IVW	0.23	0.014	0.48	NA
NPW[c]	7	IVW	0.09	0.014	0.00	0.02
AMY2B	2	IVW	0.19	0.015	0.03	NA
IL6R	15	IVW	0.03	0.016	0.00	0.09
GSN	6	IVW	−0.06	0.016	0.03	0.98
TBL2	4	IVW	−0.07	0.016	0.08	0.98
TMEM9	18	IVW	−0.04	0.018	0.01	0.43
LAMC2	11	IVW	−0.03	0.018	0.04	0.58
TDGF1[c]	19	IVW	−0.03	0.019	0.00	0.01
PPIE	7	IVW	0.07	0.020	0.03	0.96
CCN3	3	IVW	−0.09	0.020	0.03	NA
EFEMP1	13	IVW	0.05	0.020	0.01	0.42
B3GLCT	3	IVW	−0.09	0.020	0.03	NA
ESD	3	IVW	−0.04	0.021	0.09	NA
TNFRSF14	9	IVW	−0.07	0.021	0.03	0.80
ACP5	6	IVW	0.05	0.021	0.04	0.88
TEK	9	IVW	−0.05	0.021	0.04	0.71
SEMA3E	9	IVW	−0.04	0.022	0.02	0.90

WARS1	2	IVW	0.12	0.022	0.19	NA
TIE1	1	Wald ratio	−0.18	0.022	0.09	NA
QPCT	6	IVW	0.06	0.023	0.03	0.51
FABP6[ac]	9	IVW	0.08	0.024	0.01	0.01
CNTN2	9	IVW	0.05	0.025	0.00	0.26
PPT1	6	IVW	0.05	0.026	0.01	0.76
MBL2	10	IVW	0.04	0.027	0.02	0.66
CPA4	11	IVW	−0.03	0.027	0.01	0.18
LY86	3	IVW	−0.08	0.027	0.03	NA
CCL16	2	IVW	0.05	0.028	0.06	NA
MCEE	5	IVW	−0.05	0.031	0.01	1.00
IFNAR1	6	IVW	−0.05	0.031	0.02	0.55
NQO1	14	IVW	0.03	0.032	0.03	0.18
ICAM4	1	Wald ratio	0.32	0.033	0.09	NA
TRIL	2	IVW	−0.08	0.034	0.03	NA
ADAMTS5	12	IVW	−0.03	0.034	0.01	0.69
THBS3	2	IVW	0.08	0.034	0.00	NA
BTN3A1	24	IVW	−0.02	0.035	0.00	0.83
MXRA7	6	IVW	−0.12	0.037	0.10	0.18
TNFRSF10B	5	IVW	0.09	0.038	0.02	0.24
FKBP7	12	IVW	0.04	0.038	0.01	0.95
AKR1B1	1	Wald ratio	0.23	0.039	0.06	NA
TAPBP	4	IVW	−0.07	0.039	0.00	0.62
BCHE	2	IVW	0.12	0.040	0.02	NA
RSPO4[a]	1	Wald ratio	0.15	0.041	0.06	NA
CST5	11	IVW	−0.04	0.041	0.02	0.15
MAX	1	Wald ratio	0.17	0.042	0.05	NA

IL15RA	3	IVW	−0.08	0.045	0.02	NA
INSR	2	IVW	0.24	0.045	0.07	NA
IL10RB	6	IVW	−0.04	0.047	0.01	0.92
LILRA4	6	IVW	−0.07	0.048	0.02	0.21
FAH	2	IVW	0.11	0.048	0.03	NA
B3GNT8	5	IVW	−0.03	0.049	0.02	0.99
F11	3	IVW	−0.08	0.049	0.02	NA
SIGLEC7	7	IVW	0.03	0.050	0.03	0.99
MAN2B2	6	IVW	−0.02	0.050	0.01	0.65

[a] PD protein marker in Supplementary Table 3
[b] passed FDR correction
[c] corrected by removing outlier by MRPRESSO
[d] has homolog detected by the same SOMAmer

MAF>0.05, cis-pQTL p<5e-08, F-statistic>10, clumped by r2<0.3 for SNPs within 1000kb region
744 proteins have a significant cis-pQTL

Supplementary Table 3 Mendelian randomization using high LD threshold

Protein	Nr of IVs	Method	β	Nominal *P*-value	Colocalization PP.H4.abf
CD38[a]	1	Wald ratio	−0.53	2.62×10^{-11}	0.45
GPNMB[a,b]	1	Wald ratio	0.15	1.47×10^{-8}	0.94
HLA-DQA2[a]	2	IVW	−0.21	1.93×10^{-7}	0.03
FCGR2A[a]	1	Wald ratio	0.06	4.37×10^{-5}	0.95
ASIP	2	IVW	−0.14	7.99×10^{-4}	0.17
CTSB	1	Wald ratio	−0.10	0.0011	0.20
ARSA[b]	1	Wald ratio	0.15	0.0012	0.58
TPSB2	2	IVW	−0.06	0.0015	0.15
NCR1	1	Wald ratio	0.19	0.0015	0.39
LRP12[b]	1	Wald ratio	0.73	0.0017	0.66
HAVCR2	2	IVW	−0.09	0.0027	0.30
CLN5[b]	1	Wald ratio	−0.13	0.0033	0.38
CLEC3B	1	Wald ratio	−0.15	0.0035	0.29
ICAM1	1	Wald ratio	−0.07	0.0037	0.32
DSCAM	1	Wald ratio	−0.22	0.0044	0.19
PGD	1	Wald ratio	0.17	0.0052	0.48
ADAMTS4	1	Wald ratio	−0.07	0.0058	0.19
WARS1	1	Wald ratio	0.16	0.0060	0.19
FGFRL1	1	Wald ratio	0.14	0.0071	0.00
GRN[b]	1	Wald ratio	−0.19	0.0097	0.22
EPHA1	2	IVW	0.06	0.010	0.08
SIGLEC6	1	Wald ratio	0.27	0.014	0.19
AGT	1	Wald ratio	0.14	0.015	0.06
PAM	1	Wald ratio	0.09	0.015	0.08
LTF	1	Wald ratio	0.04	0.016	0.04
PLXNB2[b]	1	Wald ratio	−0.03	0.019	0.08
A4GALT	1	Wald ratio	−0.17	0.019	0.11
TXNDC15	1	Wald ratio	−0.05	0.020	0.12
EGF	1	Wald ratio	−0.11	0.021	0.11
HRG	1	Wald ratio	−0.09	0.021	0.09
TIE1	1	Wald ratio	−0.18	0.022	0.09

BST1	2	IVW	0.10	0.023	0.24
RABEPK	1	Wald ratio	−0.05	0.023	0.10
MXRA7	1	Wald ratio	−0.14	0.024	0.10
GLCE	1	Wald ratio	0.04	0.025	0.10
S100A7	1	Wald ratio	0.08	0.026	0.16
ESD	1	Wald ratio	−0.05	0.030	0.09
CCN3	2	IVW	−0.09	0.032	0.03
MANEA	3	IVW	0.04	0.032	0.10
ICAM4	1	Wald ratio	0.32	0.033	0.09
FBP2	1	Wald ratio	−0.17	0.035	0.05
CCL15	1	Wald ratio	−0.07	0.037	0.12
PLA2G7	1	Wald ratio	0.05	0.039	0.05
AKR1B1	1	Wald ratio	0.23	0.039	0.06
IDUA	1	Wald ratio	0.05	0.039	0.00
HP	2	IVW	0.05	0.039	0.01
C1QL1	2	IVW	0.10	0.040	0.00
CCL23	2	IVW	0.08	0.040	0.03
RSPO4[b]	1	Wald ratio	0.15	0.041	0.06
MAX	1	Wald ratio	0.17	0.042	0.05
ENTPD6	2	IVW	−0.15	0.044	0.11
CLEC7A	1	Wald ratio	0.04	0.045	0.09
INSR	2	IVW	0.24	0.045	0.07
TMPRSS5	1	Wald ratio	−0.07	0.047	0.06
H6PD	1	Wald ratio	−0.08	0.049	0.05

[a] passed FDR correction
[b] PD protein marker in Supplementary Table 3

MAF>0.05, cis-pQTL p<5e-08, F-statistic>10, clumped by r2<0.01 for SNPs within 1000kb region
744 proteins have a significant cis-pQTL

Supplementary Table 4 Significant differences at proteome level between PD patients (all) and controls (all)

Gene symbol	PD change direction	*P*-value (FDR adjusted)
LPO	-	2.35×10^{-08}
SEMG2	-	9.67×10^{-08}
DLK1	-	1.76×10^{-07}
RIPK2	-	3.91×10^{-07}
AMP	+	4.35×10^{-05}
NETO1	-	1.02×10^{-04}
PLXNB2	+	4.62×10^{-04}
BRICD5	-	1.05×10^{-03}
ADM	-	1.05×10^{-03}
UNC5D	-	1.05×10^{-03}
SCUBE1	+	1.05×10^{-03}
CTSO	+	1.05×10^{-03}
VEGFA	-	1.19×10^{-03}
DDR1	+	1.28×10^{-03}
GPI	+	1.64×10^{-03}
VWC2L	-	1.64×10^{-03}
CNTFR	+	1.67×10^{-03}
TMEM106A	+	1.68×10^{-03}
MDK	+	1.68×10^{-03}
VIP	-	1.76×10^{-03}
ETS2	+	2.16×10^{-03}
SHANK1	-	2.16×10^{-03}
PTPRR	-	2.20×10^{-03}
MAN1C1	+	2.63×10^{-03}
SEMA6A	+	2.63×10^{-03}
HS6ST1	+	3.66×10^{-03}
TENM4	-	5.51×10^{-03}
FRZB	+	7.48×10^{-03}
ARSA	+	7.58×10^{-03}
DPP7	+	7.71×10^{-03}
FAM171B	+	7.80×10^{-03}
CREG1	+	7.80×10^{-03}
OCRL	-	8.08×10^{-03}
CNTN1	+	8.08×10^{-03}
PRDX3	+	8.08×10^{-03}
DPYSL5	+	8.70×10^{-03}
EPHA5	-	8.70×10^{-03}
IL17D	+	8.70×10^{-03}
CHST5	+	8.70×10^{-03}
FZD10	-	8.83×10^{-03}
ARTN	-	9.03×10^{-03}
EFNB3	-	9.05×10^{-03}
SMIM24	-	0.0101
NPTX2	-	0.0106
LRP12	-	0.0106
CD109	+	0.0112
IFNGR1	+	0.0116
CDH1	+	0.0117
GCH1	+	0.0121
CCL14	+	0.0121
AK1	+	0.0121
SPINK9	-	0.0121
GXYLT1	+	0.0121

NEFH	+	0.0121
HGF	+	0.0147
ATP1B2	+	0.0166
TFPI	+	0.0169
FTH1 / FTL	+	0.0169
MAPK1	+	0.0180
TREM2	+	0.0180
GPNMB	+	0.0185
MEGF10	-	0.0187
CPE	+	0.0187
NIPAL4	+	0.0201
TOPBP1	-	0.0201
PLOD3	+	0.0220
CLN5	+	0.0227
RSPO4	-	0.0228
CDCP1	+	0.0258
PTN	+	0.0261
OMD	+	0.0261
NFASC	+	0.0261
SMPD1	+	0.0274
MMP8	-	0.0274
CHST6	+	0.0274
CEL	+	0.0278
GNS	+	0.0304
C1QTNF1	+	0.0311
CA10	+	0.0325
IL10	-	0.0325
CD209	-	0.0325
ACP2	+	0.0325
ROBO3	-	0.0331
RAF1	-	0.0354
SLITRK3	+	0.0359
PCSK1	-	0.0359
CTSC	+	0.0359
CECR1	+	0.0359
NDUFB4	-	0.0363
SERPINB1	+	0.0363
TGFBR3	+	0.0363
IGF1R	+	0.0363
GZMB	+	0.0365
ADAM9	+	0.0365
ADAM22	+	0.0370
AP2A2	-	0.0381
GRN	+	0.0381
ADCYAP1	-	0.0383
CD44	-	0.0390
CLMP	+	0.0390
RELT	-	0.0393
MATN2	+	0.0402
CD2	+	0.0402
PCDH9	+	0.0402
B3GALT2	-	0.0402
PSMB5	+	0.0402
DNAJB11	-	0.0402
KIAA1549L	+	0.0426
GGH	+	0.0437

BCAN	+	0.0454
SIAE	+	0.0454
GRAMD1C	-	0.0455
LIFR	+	0.0465
GRB2	-	0.0470
TRAPPC4	+	0.0470
SMURF1	-	0.0473
CTSH	+	0.0473
EPHB6	-	0.0490
LARGE1	+	0.0490
LYVE1	-	0.0494
BTC	+	0.0494

The change in PD represents (+) increased and (-) decreased in Parkinson's disease patients vs controls, respectively. Note**:** there are 122 proteins tagged by 129 SOMAmers. Duplicated SOMAmers are removed from the table for clarity (the one with the lowest *P*-value is kept). No discrepancies in directionality were present for duplicated SOMAmers.

Supplementary Table 5 Significant differences at proteome level between endotypes 1 and 2 of the Parkinson's disease idiopathic subcohort and healthy controls

Gene symbol	PD change direction	*P*-value (FDR adjusted)
**Endotype 1 vs HC**		
CNTFR	+	0.0178
LPO	-	5×10^{-06}
MMP10	-	0.0178
RIPK2	-	0.0178
VEGFA	-	0.0378
**Endotype 2 vs HC**		
ACVR2B	+	0.0302
ADAM10	-	0.0289
ADGRF5	-	0.0113
ADH4	-	0.0260
ADI1	-	0.0087
ADM	-	0.0152
AGA	-	0.0322
AK1	+	0.0039
ANGPTL7	-	0.0128
ANTXR1	-	0.0443
APBB2	-	0.0390
ARHGAP30	-	0.0184
ASCC1	-	0.0336
ATRAID	-	0.0461
BCAN	+	0.0266
BHMT2	-	0.0185
BMPR1A	+	0.0152
BMPR1B	+	0.0282
BRICD5	-	0.0268
BTC	+	0.0328
C1RL	-	0.0378
C4BPB	-	0.0398
CACYBP	-	0.0253
CALB1	-	0.0016
CBLN4	-	0.0339
CBS	-	0.0113
CCL14	+	0.0184
CCL26	+	0.0113
CD109	+	0.0103
CDC42BPA	-	0.0311
CDH1	+	0.0113
CELA3B	-	0.0111
CES1	-	0.0103
CHFR	-	0.0367
CLPSL2	+	0.0291
COL15A1	+	0.0087
COMP	-	0.0185
COX4I2	+	0.0291
CPE	+	0.0339
CRIM1	+	0.0039
CRTAC1	-	0.0122
CYCS	-	0.0157

DAG1	+	0.0121
DCTN2	-	0.0151
DEFA5	-	0.0444
DGKB	-	0.0121
DLK2	-	0.0339
DNER	+	0.0268
DUSP6	-	0.0256
EDA	+	0.0493
EFNB3	-	0.0260
EFS	-	0.0303
EMID1	+	0.0183
ENTPD5	-	0.0339
EPHA3	-	0.0311
EPHA4	-	0.0157
EPHA5	-	0.0087
EPHB6	-	0.0468
FAF2	+	0.0363
FAP	-	0.0113
FCN3	-	0.0039
FGF12	-	0.0215
FGF7	+	0.0495
FGL1	-	0.0435
FRZB	+	0.0103
GABBR1	-	0.0089
GDF11	-	0.0443
GFRA2	-	0.0378
GP1BA	-	0.0296
GPI	+	0.0039
GRB2	-	0.0269
GSTA1	+	0.0138
HAMP	+	0.0033
HAPLN1	-	0.0067
HCAR2	-	0.0113
HEPACAM2	-	0.0450
HGF	+	0.0260
HIKESHI	+	0.0432
HLA-DMA	-	0.0311
HPGD	-	0.0303
HS6ST1	+	0.0334
ICAM2	-	0.0141
IGFBP2	+	0.0113
IGFBPL1	-	0.0172
IGLL1	-	0.0289
IL17D	+	0.0157
IL19	-	0.0387
IL27RA	-	0.0018
ITSN1	-	0.0138
JAG1	-	0.0039
KAT6A	+	0.0157
KERA	+	0.0103
LIPN	-	0.0435
LPO	-	0.0068
LRFN2	-	0.0071
LRP12	-	0.0138
LYVE1	-	0.0432
MAN1B1	-	0.0119

MAN1C1	+	0.0131
MATN2	+	0.0032
MDK	+	0.0087
MEGF10	-	0.0151
MFNG	-	0.0458
MINOS1	-	0.0496
MLEC	+	0.0183
MMP8	-	0.0243
MPZ	+	0.0493
MST1R	+	0.0067
NEFH	+	0.0222
NELL2	+	0.0241
NETO1	-	0.0021
NPTN	-	0.0157
NPTX2	-	0.0113
NRXN1	-	0.0113
NXPH3	-	0.0151
OCIAD1	-	0.0303
OCRL	-	0.0154
OMD	+	0.0115
OXT	+	0.0112
PAPPA2	+	0.0311
PGD	+	0.0260
PKN1	-	0.0289
PLA2G2C	+	0.0113
PLOD3	+	0.0087
PLXNA1	-	0.0087
PPA1	-	0.0089
PRDM4	-	0.0346
PRG2	-	0.0183
PRRG4	-	0.0379
PSMD5	-	0.0244
PTPRR	-	0.0036
PZP	-	0.0328
RAB31	-	0.0447
RAB6B	-	0.0121
RAF1	-	0.0103
RAPGEF5	+	0.0468
RB1	+	0.0121
RBL2	-	0.0333
RBP4	-	0.0291
RELT	-	0.0334
RIPK2	-	0.0014
RNASE4	+	0.0138
ROBO3	-	0.0089
ROR1	-	0.0336
RSPO4	-	0.0445
SCARA5	-	0.0499
SCG2	-	0.0119
SCUBE1	+	0.0005
SERPINA10	-	0.0151
SERPINA9	-	0.0014
SFTA2	-	0.0087
SHANK1	-	0.0018
SHANK3	-	0.0224
SMIM24	-	0.0103

SMURF1	-	0.0291
SOD3	+	0.0218
SPINK9	-	0.0138
SPOCK3	+	0.0131
SPSB1	+	0.0497
SRXN1	-	0.0113
ST6GALNAC6	+	0.0260
STMN3	-	0.0157
STX10	-	0.0014
STX12	-	0.0103
STX1A	-	0.0039
STX1B	-	0.0398
STX2	-	0.0291
STX3	-	0.0322
STX7	-	0.0132
SV2A	-	0.0456
TCN2	+	0.0339
TESC	-	0.0365
TFPI	+	0.0164
TFRC	+	0.0157
TGFB3	-	0.0390
THBS2	+	0.0039
TMEM132B	-	0.0443
TMEM132D	-	0.0157
TMEM230	-	0.0260
TMEM8B	-	0.0486
TNFRSF11B	+	0.0121
TNFRSF13C	-	0.0339
TNFRSF18	-	0.0260
TNFRSF1A	+	0.0289
TPO	+	0.0289
TPSG1	+	0.0205
TYMP	-	0.0138
UBL4A	-	0.0113
UNC5D	-	0.0121
USE1	-	0.0113
VEGFA	-	0.0301
VIP	-	0.0103
VWC2	-	0.0260
VWC2L	-	0.0028
WFDC13	+	0.0483
YWHAG	-	0.0117
YWHAZ, YWHAE, YWHAG, YWHAH, SFN, YWHAQ, YWHAB	-	0.0486
ZNF10	+	0.0339

Endotype 1 vs Endotype 2

ADAM10	+	0.0141
ADAM23	+	0.0106
ADAMTSL2	+	0.0210
ADGRF5	+	0.0497
ADM	+	0.0480
AGA	+	0.0325
AKT2	-	0.0323

ANGPTL7	+	0.0088
APBB2	+	0.0462
ASIC4	-	0.0277
ATP5B	+	0.0465
AXIN2	-	0.0497
B4GALT7	-	0.0348
BAG4	+	0.0235
BDNF	+	0.0325
BMPR1B	-	0.0135
BMPR2	+	0.0274
BRD2	-	0.0421
C1QBP	+	0.0385
C4BPB	+	0.0480
CA4	+	0.0323
CA5A	+	0.0309
CACYBP	+	0.0403
CALB1	+	0.0305
CAPN2	+	0.0462
CASS4	+	0.0309
CCL26	-	0.0262
CD300E	+	0.0467
CD47	+	0.0048
CD96	+	0.0130
CDC42BPA	+	0.0391
CELA3B	+	0.0106
CES1	+	0.0048
CHFR	+	0.0049
CHP1	+	0.0323
CKS1B	+	0.0355
CLCA1	+	0.0411
CLEC2L	+	0.0309
CNTFR	+	0.0231
COX4I2	-	0.0176
CRB1	-	0.0480
CRIM1	-	0.0106
CTSH	+	0.0412
CUL4B	+	0.0160
CXCL2, CXCL3	+	0.0325
CYB5R3	+	0.0106
CYP3A4	+	0.0480
DCTN2	+	0.0323
DGKB	+	0.0274
DLL4	+	0.0409
DNAJB12	-	0.0323
DUSP26	+	0.0326
DUSP6	+	0.0298
EIF4G3	+	0.0391
EMID1	-	0.0256
FAP	+	0.0210
FBLN5	-	0.0325
FCN3	+	0.0336
FJX1	-	0.0374
FLRT2	-	0.0334
FSTL1	-	0.0467
GABBR1	+	0.0088
GALNT11	-	0.0325

GP1BA	+	0.0336
GPD1	+	0.0274
GRB14	+	0.0323
HCAR2	+	0.0394
HLA-DMA	+	0.0277
HPGD	+	0.0391
ICAM2	+	0.0105
IFNG	+	0.0351
IFNGR2	+	0.0176
IGFBPL1	+	0.0274
IGLL1	+	0.0296
IL1B	-	0.0088
IL22	+	0.0323
IL27RA	+	0.0106
ILF3	+	0.0482
ITSN1	+	0.0041
KAT6A	-	0.0323
KEAP1	+	0.0325
KIRREL3	+	0.0412
KLRC4	+	0.0391
LECT1	+	0.0391
LIPN	+	0.0325
LRFN2	+	0.0374
LTBP4	-	0.0355
LYG1	+	0.0467
MADCAM1	+	0.0465
MAN1B1	+	0.0355
MDK	-	0.0467
MINOS1	+	0.0176
MRM3	+	0.0306
MST1R	-	0.0268
MTMR1	-	0.0262
MYRF	-	0.0339
NAT14	+	0.0480
NDUFV2	+	0.0305
NEGR1	+	0.0235
NPTN	+	0.0048
NRXN1	+	0.0325
NTM	+	0.0274
NXPH3	+	0.0202
PAPPA2	-	0.0176
PELO	+	0.0296
PLOD3	-	0.0165
PPA1	+	0.0088
PPBP	+	0.0480
PRDM4	+	0.0157
PRG2	+	0.0348
PYDC1	+	0.0410
PZP	+	0.0456
RB1	-	0.0295
RBBP6	+	0.0480
RBL2	+	0.0497
RNF8	+	0.0262
ROBO2	+	0.0482
ROR2	-	0.0262
RYK	+	0.0391

SCARA5	+	0.0467
SCARB2	+	0.0391
SCG2	+	0.0325
SCN2B	+	0.0165
SCUBE1	-	0.0130
SERPINA10	+	0.0480
SERPINA9	+	0.0106
SGF29	+	0.0355
SLC14A2	+	0.0119
SOD2	+	0.0277
SPOCK3	-	0.0325
ST6GALNAC6	-	0.0305
STAB2	-	0.0467
STMN3	+	0.0348
STX10	+	0.0007
STX12	+	0.0193
STX1A	+	0.0235
STX7	+	0.0059
TESC	+	0.0259
TFRC	-	0.0274
THBS2	-	0.0325
THYN1	+	0.0325
TMEM52B	+	0.0421
TMEM8B	+	0.0325
TNFRSF18	+	0.0274
TP63	+	0.0165
TPO	-	0.0391
TPST1	-	0.0275
TRAPPC5	-	0.0176
TYMP	+	0.0077
UBTD2	+	0.0077
VTA1	-	0.0339
XG	+	0.0409
YWHAG	+	0.0274

The change in PD represents (+) increased and (-) decreased in the corresponding endotype vs controls, and endotype 1 vs endotype 2 respectively. Note**:** there are 198 proteins tagged by 200 SOMAmers for the contrast Endotype 2 vs HC and 153 proteins tagged by 155 SOMAmers for the contrast Endotype 1 vs 2. Duplicated SOMAmers are removed from the table for clarity (the one with the lowest *P*-value is kept). No discrepancies in directionality were present for duplicated SOMAmers. Proteins separated by comma in the same row represent the fact that due to lack of specificity the same SOMAmer is tagging more than one protein.

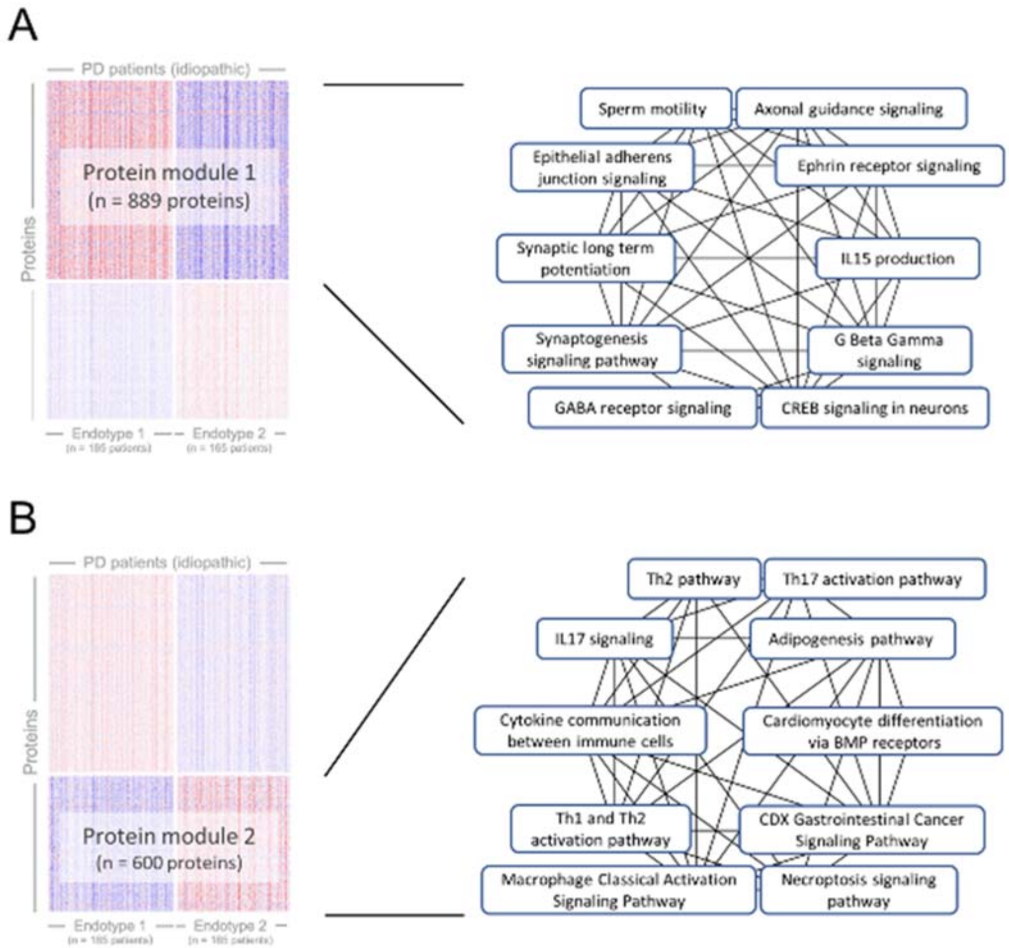

Supplementary Figure 1. Pathway Analysis generated with Ingenuity Pathway Analysis for A: protein module 1 from the heatmap of z-scores of the protein values as measured with SomaScan, corresponding to the two modules identified using Weighted Gene Co-expression Network Analysis (WGCNA) and for B: protein module 2. Lines connecting pathways represent the presence of common proteins shared by both pathways in the nodes.
