# Supplementary material for: A proteogenomic view of Parkinson’s disease causality and heterogeneity
Source: NPJ Parkinsons Dis. 2023 Feb 11;9:24. doi: 10.1038/s41531-023-00461-9 (PMC9922273; doi:10.1038/s41531-023-00461-9)
Supplement: Supplementary file 2 — Reporting Summary [file 41531_2023_461_MOESM2_ESM.pdf]

nature portfolio

Corresponding author(s): Mirko Messa
Pablo Serrano-Fernandez

Last updated by author(s): Jan 17, 2023

Reporting Summary

Nature Portfolio wishes to improve the reproducibility of the work that we publish. This form provides structure for consistency and transparency in reporting. For further information on Nature Portfolio policies, see our Editorial Policies and the Editorial Policy Checklist.

Statistics

For all statistical analyses, confirm that the following items are present in the figure legend, table legend, main text, or Methods section.

n/a	Confirmed	
☐	☒	The exact sample size (*n*) for each experimental group/condition, given as a discrete number and unit of measurement
☒	☐	A statement on whether measurements were taken from distinct samples or whether the same sample was measured repeatedly
☐	☒	The statistical test(s) used AND whether they are one- or two-sided<br>*Only common tests should be described solely by name; describe more complex techniques in the Methods section.*
☐	☒	A description of all covariates tested
☐	☒	A description of any assumptions or corrections, such as tests of normality and adjustment for multiple comparisons
☐	☒	A full description of the statistical parameters including central tendency (e.g. means) or other basic estimates (e.g. regression coefficient) AND variation (e.g. standard deviation) or associated estimates of uncertainty (e.g. confidence intervals)
☐	☒	For null hypothesis testing, the test statistic (e.g. *F*, *t*, *r*) with confidence intervals, effect sizes, degrees of freedom and *P* value noted<br>*Give P values as exact values whenever suitable.*
☒	☐	For Bayesian analysis, information on the choice of priors and Markov chain Monte Carlo settings
☒	☐	For hierarchical and complex designs, identification of the appropriate level for tests and full reporting of outcomes
☒	☐	Estimates of effect sizes (e.g. Cohen's *d*, Pearson's *r*), indicating how they were calculated

Our web collection on statistics for biologists contains articles on many of the points above.

Software and code

Policy information about availability of computer code

Data collection	The data used for this study is publicly available in the PPMI web page https://www.ppmi-info.org/access-data-specimens/download-data
Data analysis	R version 3.4, R packages: sva v3.40.0, MatrixEQTL v2.3, TwoSampleMR v0.5.6, MRPRESSO v1.0, coloc v5.1.0, limma v3.48.3, rpart v4.1.16, WGCNA v1.70.3, ConsensusClusterPlus v1.56.0, Heatplus v3.0.0

For manuscripts utilizing custom algorithms or software that are central to the research but not yet described in published literature, software must be made available to editors and reviewers. We strongly encourage code deposition in a community repository (e.g. GitHub). See the Nature Portfolio guidelines for submitting code & software for further information.

Data

Policy information about availability of data

All manuscripts must include a data availability statement. This statement should provide the following information, where applicable:

- Accession codes, unique identifiers, or web links for publicly available datasets
- A description of any restrictions on data availability
- For clinical datasets or third party data, please ensure that the statement adheres to our policy

The data used for this study is publicly available in the PPMI web page https://www.ppmi-info.org/access-data-specimens/download-data. The free access requires registration. The clinical data snapshot used here is kept under the tab “Archived PPMI data” >> “Publication Associated Archives” >> “2022-0001 Serrano-Fernandez: Parkinson's Disease Proteogenomics (Version: 2022-05-18)”. The proteomic data is available under “Biospecimen” >> “Proteomic Analysis” >> “Project

151 Identification of proteins & protein networks & pQTL analysis in CSF x of 7 (Batch Corrected)" (7 files in total). The original adat files are also available under "Biospecimen" >> "Proteomic Analysis" >> "Project 151 Identification of proteins & protein networks & pQTL analysis in CSF - ADAT files".

Human research participants

Policy information about studies involving human research participants and Sex and Gender in Research.

Reporting on sex and gender

Sex was determined based on self-report and merely used as a covariate where appropriate since this is not a sex-based analysis. The proportions of sexes in each of the subgroups in shown in figure 1. No individual-level data is shown.

Population characteristics

Covariate-relevant population characteristics are age, sex, genotypic status of the genes GBA and LRRK2 as level as treatment status.

Recruitment

Study protocol and manuals are available online (http://www.ppmi-info.org/study-design) (https://clinicaltrials.gov/ct2/show/NCT04477785.) In PPMI 2.0 Clinical up to 4,500 participants will be enrolled and followed longitudinally from approximately 40-50 international clinical sites across a variety of cohorts, including healthy controls, Parkinson disease, PD manifesting gene carriers, and Prodromal (those at risk for developing PD). Criteria:
Inclusion Criteria:
Parkinson Disease (PD) Subjects:
Male or female age 30 years or older at Screening Visit.
A diagnosis of Parkinson disease for 2 years or less at Screening Visit.
Not expected to require PD medication with at least 6 months from Baseline.
Patients must have at least two of the following: resting tremor, bradykinesia, rigidity (must have either resting tremor or bradykinesia); OR either asymmetric resting tremor or asymmetric bradykinesia.
Hoehn and Yahr stage I or II at Baseline.
Individuals taking any of the following drugs: alpha methyldopa, methylphenidate, amphetamine derivatives or modafinil, must be willing and medically able to hold the medication for at least 5 half-lives before DaTscan imaging.
Confirmation that participant is eligible based on Screening DaTscan imaging.
Able to provide informed consent
Woman may not be pregnant, lactating or planning pregnancy during the study. ~Including a negative pregnancy test on day of Screening DaTscan imaging test prior to injection of DaTscan.

Healthy Control (HC) Subjects:
Male or female age 30 years or older at Screening visit.
Individuals taking any of the following drugs: alpha methyldopa, methylphenidate, amphetamine derivatives or modafinil, must be willing and medically able to hold the medication for at least 5 half-lives before DaTscan imaging.
Confirmation that participant is eligible based on Screening DaTscan imaging.
Able to provide informed consent
Women may not be pregnant, lactating or planning pregnancy during the study. ~ Includes a negative pregnancy test on day of Screening DaTscan imaging test prior to injection of DaTscan™.
Exclusion Criteria:

Parkinson Disease (PD) Subjects:

Currently taking levodopa, dopamine agonists, MAO-B inhibitors (e.g., selegiline, rasagiline), amantadine or other PD medication.
Has taken levodopa, dopamine agonists, MAO-B inhibitors or amantadine within 60 days of Baseline.
Has taken levodopa or dopamine agonists prior to Baseline for more than a total of 90 days.
Atypical PD syndromes due to either drugs (e.g., metoclopramide, flunarizine, neuroleptics) or metabolic disorders (e.g., Wilson's disease), encephalitis, or degenerative diseases (e.g., progressive supranuclear palsy)
A clinical diagnosis of dementia as determined by the investigator.
Previously obtained MRI scan with evidence of clinically significant neurological disorder (in the opinion of the Investigator)
Received any of the following drugs: dopamine receptor blockers (neuroleptics), metoclopramide and reserpine within 6 months of Screening visit.
Current treatment with anticoagulants (e.g. coumadin, heparin, oral thrombin inhibitors) that might preclude safe completion of lumbar puncture.
Condition that precludes the safe performance of routine lumbar puncture, such as prohibitive lumbar spinal disease, bleeding diathesis, or clinically significant coagulopathy or thrombocytopenia.
Any other medical or psychiatric condition or lab abnormality, which in the opinion of the investigator might preclude participation.

Healthy Control (HC) Subjects:

Current or active clinically significant neurological disorder (in the opinion of the Investigator).
First degree relative with PD (parent, sibling, child).
Previously obtained MRI scan with evidence of clinically significant neurological disorder (in the opinion of the Investigator)
Received any of the follow drugs: dopamine receptor blockers (neuroleptics), metoclopramide and reserpine within 6 months of Screening visit.
Current treatment with anticoagulants (e.g., coumadin, heparin, oral thrombin inhibitors) that might preclude safe completion of the lumbar puncture.
Condition that precludes the safe performance of routine lumbar puncture, such as prohibitive lumbar spinal disease,

bleeding diathesis, or clinically significant coagulopathy or thrombocytopenia.
Any other medical or psychiatric condition or lab abnormality, which in the opinion of the investigator might preclude participation.
Inclusion Criteria:

(PD-LRRK2 or GBA) Participants:

Male or female age 30 years or older at Screening visit.
A diagnosis of Parkinson disease for 2 years or less at Screening Visit.
Patients must have a least two of the following: resting tremor, bradykinesia, rigidity (must have either resting tremor or bradykinesia); OR either asymmetric resting tremor or asymmetric bradykinesia.
Hoehn and Yahr stage I or II at Baseline.
Confirmation of causative LRRK2 or GBA (willingness to undergo genetic testing as part of genetic screening and be informed of genetic testing results, or documentation of prior genetic testing results).
Individuals taking any of the following drugs: alpha methyldopa, methylphenidate, amphetamine derivatives or modafinil, must be willing and medically able to hold the medication for at least 5 half-lives before DaTscan imaging.
Confirmation that participant is eligible based on Screening DaTscan imaging.
Able to provide informed consent
Woman may not be pregnant, lactating or planning pregnancy during the study. ~Including a negative pregnancy test on day of Screening DaTscan imaging test prior to injection of DaTscan™.
Exclusion Criteria:

PD-LRRK2 or GBA

Received any of the following drugs: dopamine receptor blockers (neuroleptics), metoclopramide and reserpine within 6 months of Screening visit.
Current treatment with anticoagulants (e.g., coumadin, heparin) that might preclude safe completion of the lumbar puncture
Condition that precludes the safe performance of routine lumbar puncture, such as prohibitive lumbar spinal disease, bleeding diathesis, or clinically significant coagulopathy or thrombocytopenia.
Any other medical or psychiatric condition or lab abnormality, which in the opinion of the investigator might preclude participation.

Ethics oversight

PPMI samples were collected under a standardized protocol over 33 centers. The study was approved by the Institutional Review Board at each site, and participants provided written informed consent.

Note that full information on the approval of the study protocol must also be provided in the manuscript.

Field-specific reporting

Please select the one below that is the best fit for your research. If you are not sure, read the appropriate sections before making your selection.

☒ Life sciences ☐ Behavioural & social sciences ☐ Ecological, evolutionary & environmental sciences

For a reference copy of the document with all sections, see nature.com/documents/nr-reporting-summary-flat.pdf

Life sciences study design

All studies must disclose on these points even when the disclosure is negative.

Sample size

- 569 patients (350 idiopathic patients, 65 GBA+ mutation carriers and 154 LRRK2+ mutation carriers)
- 534 controls

Data exclusions

For genetics:
Standard GWAS quality control (QC) was applied at both individual and SNP level. 22 patients with outlying heterozygosity and 93 patients with high identity-by-descent were excluded after QC. 306031 SNPs were removed due to missing genotype and 35907596 SNPs removed due to minor allele count less than 20. Finally, 9743041 variants and 1264 subjects passed QC.

For proteomics:
The original data set was comprised of 1190 samples out of which 32 samples were pools, which were discarded for this study. Additional six Parkinson's disease patients and one healthy control were removed due to change in diagnose during the trial, 38 GBA+ subjects were removed because they carried non-severe GBA mutations, and 10 patients were removed for being carriers of a mutation in SCNA.

Replication

N/A

Randomization

N/A

Blinding

N/A

Reporting for specific materials, systems and methods

We require information from authors about some types of materials, experimental systems and methods used in many studies. Here, indicate whether each material, system or method listed is relevant to your study. If you are not sure if a list item applies to your research, read the appropriate section before selecting a response.

Materials & experimental systems

n/a	Involved in the study	
☒	☐	Antibodies
☒	☐	Eukaryotic cell lines
☒	☐	Palaeontology and archaeology
☒	☐	Animals and other organisms
☐	☒	Clinical data
☒	☐	Dual use research of concern

Methods

n/a	Involved in the study	
☒	☐	ChIP-seq
☒	☐	Flow cytometry
☒	☐	MRI-based neuroimaging

Clinical data

Policy information about clinical studies

All manuscripts should comply with the ICMJE guidelines for publication of clinical research and a completed CONSORT checklist must be included with all submissions.

Clinical trial registration	ClinicalTrials.gov Identifier: NCT04477785
Study protocol	http://www.ppmi-info.org/study-design<br>https://clinicaltrials.gov/ct2/show/NCT04477785
Data collection	PPMI began initial recruitment in 2010. After an enrollment hiatus, the study expanded and reinitiated recruitment in 2020. There are some differences in the protocol and eligibility criteria between these two phases. All PPMI data has been harmonized in the current data structure.
Outcomes	Scores:<br>UPDRS scores, MoCA scores, ngPD-ProS scores<br><br>CSF protein levels (clinical assay):<br>amyloid beta, phospho-tau, total tau and alpha-synuclein<br><br>Brain thickness changes:<br>caudate, putamen, striatum
